# Supplementary material for: Speedy A governs non-homologous XY chromosome desynapsis as a unique prerequisite for XY loop-axis organization
Source: EMBO J. 2025 Aug 18;44(19):5509–36. doi: 10.1038/s44318-025-00528-8 (PMC12488978; doi:10.1038/s44318-025-00528-8)
Supplement: Supplementary file 1 — Appendix [file 44318_2025_528_MOESM1_ESM.pdf]

**Appendix for**

# Speedy A governs non-homologous XY desynapsis as a unique prerequisite for XY loop-axis organization

By Dongteng Liu et al.

**Table of Contents:**

**Supplementary Result 1**. Validation of the mouse model of synchronized meiotic prophase I. ..……………...………………………...………3

**Supplementary Result 2**. Specific disruption of the *Trf1* gene in mouse pachytene spermatocytes. …………………………...…………...……3

**Supplementary Result 3**. Absence of TRF1 disrupted Tel-NE attachment, leading to the demise of pachytene spermatocytes. ……………………..…4

**Supplementary Result 4**. The mouse SpdyA$^{A125V}$ protein exhibited defective protein production and shortened half-life. ……………..……………5

**Appendix Figure S1**. Manipulation of retinoic acid signaling allows synchronous development of germ cells. ………..…………..………..……6

**Appendix Figure S2**. Normal DSB formation in the PAR and meiotic progression in *Sync-Spdya$^{cKO}$* pachytene spermatocytes. …..…………..……8

**Appendix Figure S3.** Bulk RNA-seq analysis revealed similar transcriptional profiles between *Sync-Spdya$^{fl/fl}$* and *Sync-Spdya$^{cKO}$* mid-late pachytene testes. ………………………………………………...……9

**Appendix Figure S4.** Tamoxifen-induced knockout of *Spdya* in juvenile mice leads to persistent meiotic arrest and absence of spermatozoa in adulthood. ……………………………………………..……10

**Appendix Figure S5.** Knockout of *Spdya* did not impair autosomal desynapsis. …....11

**Appendix Figure S6.** Normal X-Y loop-axis organization in late pachytene *Sync-*$Spdya^{cKO}$ and $Spdya^{A125V}$ spermatocytes with end-to-end X-Y configuration. ……12

**Appendix Figure S7.** Normal telomeric localization of TRF1, TERB1 and MAJIN on sex chromosome in early pachytene *Sync-*$Spdya^{cKO}$ spermatocytes. ……13

**Appendix Figure S8.** Deletion of the *Trf1* gene in pachytene spermatocytes in mice. ……14

**Appendix Figure S9.** Identification of a homozygous missense mutation in human *SPDYA* gene in a NOA patient. ……15

**Appendix Figure S10.** The A125V substitution reduces the stability of SpdyA protein in HEK293T cells. ……16

**Appendix Figure S11.** Normal meiotic progression and crossover formation in $Spdya^{A125V}$ pachytene spermatocytes. ……17

**Appendix Figure S12.** The p-SUN1(S48) were specifically localized at telomeres in prophase I mouse spermatocytes. ……18

**Appendix Figure S13.** Functional validation of *Ddx4-*$Cre^{ERT2}$ knockin mice using *mT/mG* reporter mice. ……19

**Appendix Table S1.** Information of the primers used for genotyping, Sanger sequencing and quantitative PCR. ……20

**Appendix Table S2.** Clinical and semen characteristics in the proband with the bi-allelic *SPDYA* variant with A126V missense mutation. ……21

**Appendix Table S3.** List of antibodies. ……22

**Appendix Reference.** ……24

## Supplementary Results

### 1. Validation of the mouse model of synchronized meiotic prophase I.

Briefly, PD2 neonatal male mouse pups were *s.c.* injected with WIN 18,446 (100 mg/kg body weight) daily for 7 consecutive days to suppress spermatogonial differentiation. At PD9, mice were *s.c.* injected with RA (12.5 mg/kg body weight) to synchronously restore spermatogenesis. To validate the state of synchronous prophase I, we collected testes at different days post RA restoration and performed immunostaining for SYCP3/SYCP1 or γH2AX/H1t on the testis sections.

The analysis provided a timeline of synchronized prophase I spermatocytes as summarized in Appendix Fig. S1A. As shown in Appendix Fig. S1B (a-a″), at RA7.25 the synchronized seminiferous tubules only contained leptotene spermatocytes without SYCP1 signals. These spermatocytes developed quickly and became zygotene cells with incomplete SYCP1 signals at RA7.75 (Appendix Fig. S1B, b-b″). At RA9.75, the seminiferous tubules contained early pachytene spermatocytes with all SYCP1 signals overlapping the SYCP3 signals on all autosomes (Appendix Fig. S1B, c-c″) along with aggregated γH2AX signals at the sex body area (Appendix Fig. S1B-c‴), while H1t signal was absent in the nucleus (Appendix Fig. S1B-c‴). At RA11.75, the synchronized tubules harbored mid-to-late pachytene spermatocytes with moderate H1t signals (Appendix Fig. S1B, d-d‴) that became much stronger at RA13.25 when the first wave of synchronized spermatocytes were in late pachytene stage (Appendix Fig. S1B, e-e‴). Note that the second wave of spermatocytes entered leptotene stage at that moment. Lastly, at RA15 and RA15.75 the first wave of synchronized spermatocytes became diplotene cells (Appendix Fig. S1B, f-f″) and metaphase I cells (Appendix Fig. S1B, g-g″).

### 2. Specific disruption of the ***Trf1*** gene in mouse pachytene spermatocytes.

The *Trf1*$^{fl/fl}$ mice were separately crossed with the germ cell-specific *Stra8-EGFPCre*

mouse line and with the tamoxifen-inducible *Ddx4-Cre*$^{ERT2}$ mouse line. The resulting fertile *Trf1*$^{fl/–}$ mice (Wang *et al*, 2018) and *Trf1*$^{fl/fl}$*;Ddx4-Cre*$^{ERT2}$ mice were then crossed with each other to generate *Trf1*$^{fl/–}$*;Ddx4-Cre*$^{ERT2}$ mice and littermate *Trf1*$^{fl/fl}$ mice. After multiple tests, we established a method to obtain conditional *Trf1*-knockout (*Trf1*$^{cKO}$) pachytene spermatocytes. Briefly, two consecutive *i.p.* injections of tamoxifen (20 mg/kg) were given to synchronized male *Trf1*$^{fl/–}$*;Ddx4-Cre*$^{ERT2}$ and *Trf1*$^{fl/fl}$ mice at RA6 and RA7 (i.e., PD15 and PD16) and their testes were collected at RA10 (i.e., PD19, illustrated in Appendix Fig. S8A).

When probed with a TRF1 antibody, the control synchronized (*Sync*)-*Trf1*$^{fl/fl}$ spermatocytes at RA10 displayed TRF1 signals at telomeres (Appendix Fig. S8B, a and a′, arrowheads), whereas the *Sync-Trf1*$^{cKO}$ pachytene spermatocytes showed a marked loss of TRF1 signals at telomeres (Appendix Fig. S8B, b and b′, arrows). Similar to *Sync-Trf1*$^{fl/fl}$ pachytene spermatocytes, the *Sync-Trf1*$^{cKO}$ cells exhibited normal pachytene SYCP1 signals in all autosomes (Appendix Fig. S8C-b, arrowheads) and aggregated γH2AX signals in the sex body region (Appendix Fig. S8C-b, arrow). Quantitative analysis revealed that 98.1% of cells in the *Sync-Trf1*$^{fl/fl}$ group and 91.6% of cells in the *Sync-Trf1*$^{cKO}$ group were pachytene spermatocytes (Appendix Fig. S8C-c).

### 3. Absence of TRF1 disrupted Tel-NE attachment, leading to the demise of pachytene spermatocytes.

As expected, compared to *Sync-Trf1*$^{fl/fl}$ early pachytene cells in which all telomeres were attached to the NE (Appendix Fig. S8D-a), *Sync-Trf1*$^{cKO}$ cells exhibited impaired Tel-NE attachment, with telomeres localized within the nuclear interior (Appendix Fig. S8D-b, arrows). As a result, *Sync-Trf1*$^{cKO}$ testes appeared smaller than *Sync-Trf1*$^{fl/fl}$ testes at RA13 (Appendix Fig. S8E-a). Histological analysis further revealed that, in contrast to *Sync-Trf1*$^{fl/fl}$ seminiferous tubules at RA13, which contained late

pachytene spermatocytes (Appendix Fig. S8E-b), *Sync-Trf1$^{cKO}$* seminiferous tubules were devoid of spermatocytes (Appendix Fig. S8E-c, asterisk). These findings suggest that TRF1 is essential for Tel-NE attachment in pachytene spermatocytes and for the survival of these cells.

## 4. The mouse SpdyA$^{A125V}$ protein exhibited defective protein production and shortened half-life.

To investigate the functional defects of the SpdyA$^{A125V}$ protein, we transfected HEK293T cells with *pcDNA3.1-Spdya$^{WT}$-HA* or *pcDNA3.1-Spdya$^{A125V}$-HA*, along with *pcDNA3.1-Cdk2-Myc* plasmids. CDK2 was consistently co-transfected with SpdyA, as it is known to stabilize SpdyA expression in transfected cells (Chen *et al*, 2021). We observed that the expression level of SpdyA$^{A125V}$-HA was markedly reduced compared to SpdyA$^{WT}$-HA in HEK293T cells (Appendix Fig. S10A).

To examine whether the lower level of SpdyA$^{A125V}$ protein was due to an enhanced proteasomal degradation via ubiquitination (Al Sorkhy *et al*, 2009), we treated the transfected cells with a proteasome inhibitor MG132. As shown in Appendix Fig. S10B, both SpdyA$^{WT}$-HA and SpdyA$^{A125V}$-HA protein levels were strongly elevated in the presence of MG132 compared to the DMSO control. However, the SpdyA$^{A125V}$-HA protein level remained consistently lower than that of SpdyA$^{WT}$-HA, suggesting that the translation/production of the SpdyA$^{A125V}$ protein *per se* may be defective.

We next examined the protein half-life. HEK293T cells were treated with cycloheximide, a protein synthesis inhibitor (Schneider-Poetsch *et al*, 2010), and harvested at various time points post-treatment. As shown in Appendix Fig. S10C, the half-life of SpdyA$^{WT}$-HA was 13.11 hours, whereas SpdyA$^{A125V}$-HA exhibited a significantly shortened half-life of 7.59 hours. These results indicate that the SpdyA$^{A125V}$ mutant protein is intrinsically less stable.

138 Appendix Figure S1

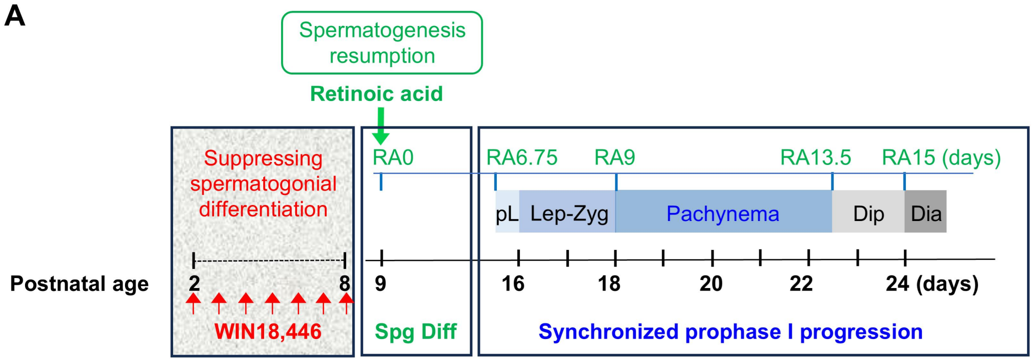


**Appendix Figure S1. Manipulation of retinoic acid signaling allows synchronous development of germ cells.**

(**A**) Illustration of synchronization procedures and timeline for synchronous development of mouse spermatocytes. PD2 neonatal male mouse pups were *s.c.* injected with WIN 18,446 (100 mg/kg body weight) for 7 consecutive days to suppress spermatogonial differentiation. At PD9, treated mice were *s.c.* injected with RA (12.5 mg/kg body weight) to restore spermatogenesis. At around RA6.75, the spermatogenic cells entered pre-leptonema and developed through leptotene and zygotene stages from RA7 to RA9. At RA9 the spermatocytes entered pachytene stage, which will last for about 4.5 days, until RA13.5. The spermatocytes then entered diplotene stage at RA13.5 and diakinesis stage at RA15.5.

(**B**) A series of immunostaining for SYCP3 (red) and SYCP1 (green) on testis sections collected at different days post RA restoration, validating the state of synchronous prophase I. L, leptotene spermatocyte; Z, zygotene spermatocyte; EP, early pachytene spermatocyte; MP, mid-pachytene spermatocyte; LP, late pachytene spermatocyte; D, diplotene spermatocyte; MI, metaphase I spermatocyte. Scale bars, 20 μm. (c‴, d‴ and e‴) H1t and γH2AX co-staining on testis sections showing that no H1t signals in early pachytene spermatocytes (RA9.75, c‴), moderate H1t signals in mid-pachytene spermatocytes (RA11.75, d‴) and strong H1t signals in late pachytene spermatocytes (RA13.25, e‴). Scale bars, 20 μm.

139 Appendix Figure S1, continued

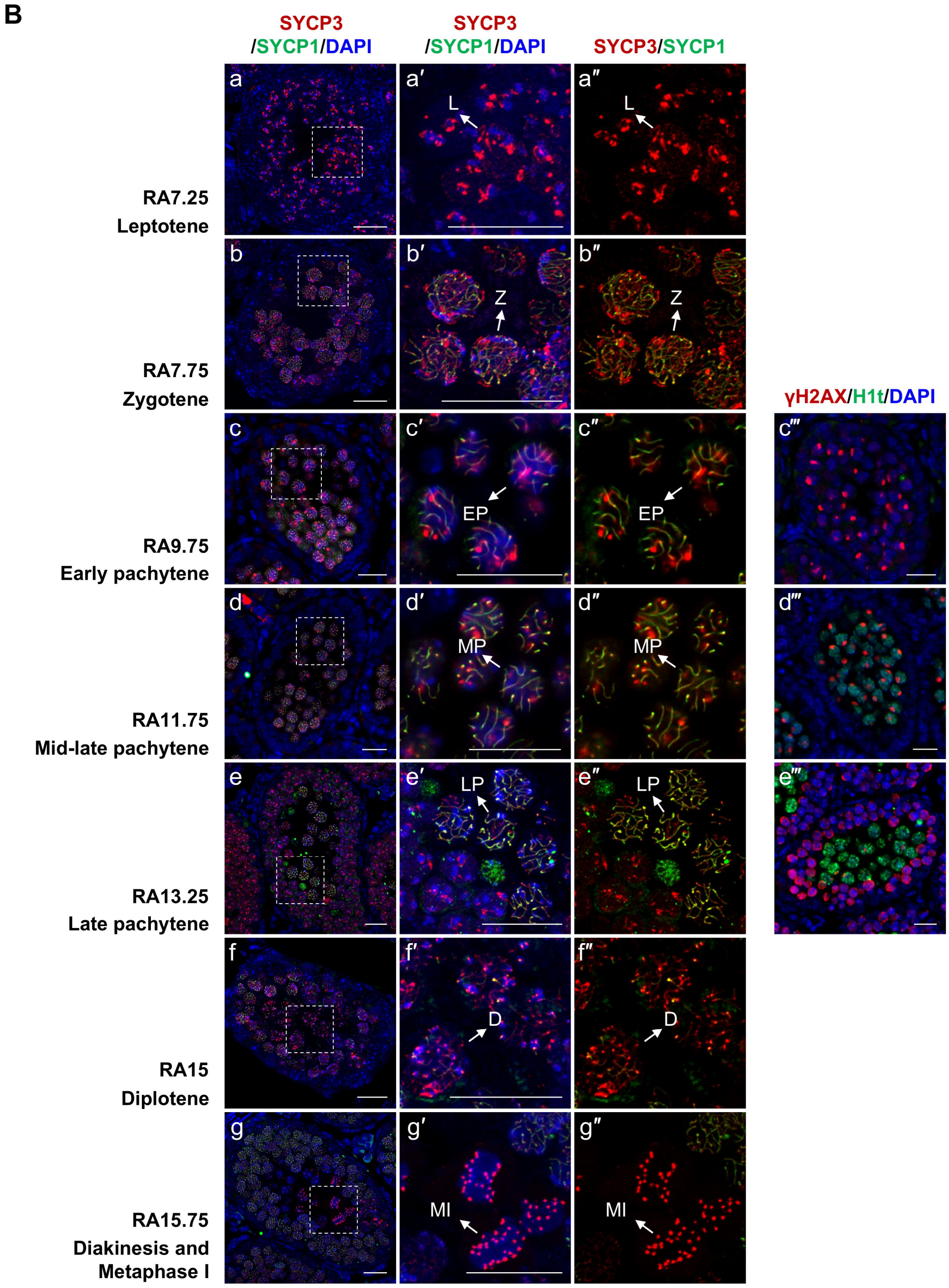

# Appendix Figure S2

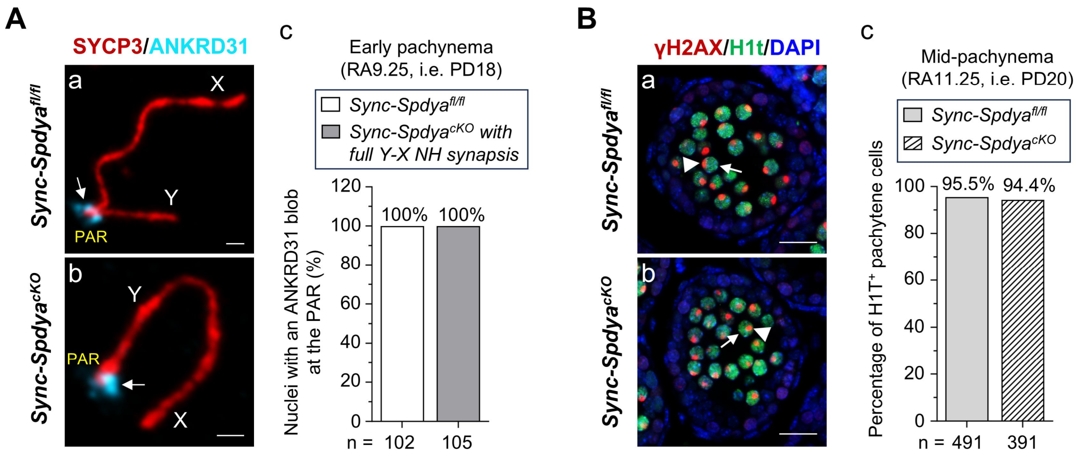


**Appendix Figure S2. Normal DSB formation in the PAR and meiotic progression in *Sync-Spdya$^{cKO}$* pachytene spermatocytes.**

(**A**) A normal ANKRD31 blob was observed at the PAR in early pachytene (RA9.25) *Sync-Spdya$^{cKO}$* cells with *full Y-X NH synapsis* (b). Scale bars, 1 μm. (c) Percentages of nuclei with an ANKRD31 blob at the PAR in each genotype.

(**B**) IF analyses of *Sync-Spdya$^{fl/fl}$* (a) and *Sync-Spdya$^{cKO}$* (b) testis sections stained for H1t (green, arrows), γH2AX (red, arrowheads) and DAPI (blue), showing whole-nucleus distribution of H1t in *Sync-Spdya$^{cKO}$* pachytene spermatocyte. Scale bars, 30 μm. (c) Percentages of H1t-positive pachytene cells in each genotype.

## Appendix Figure S3

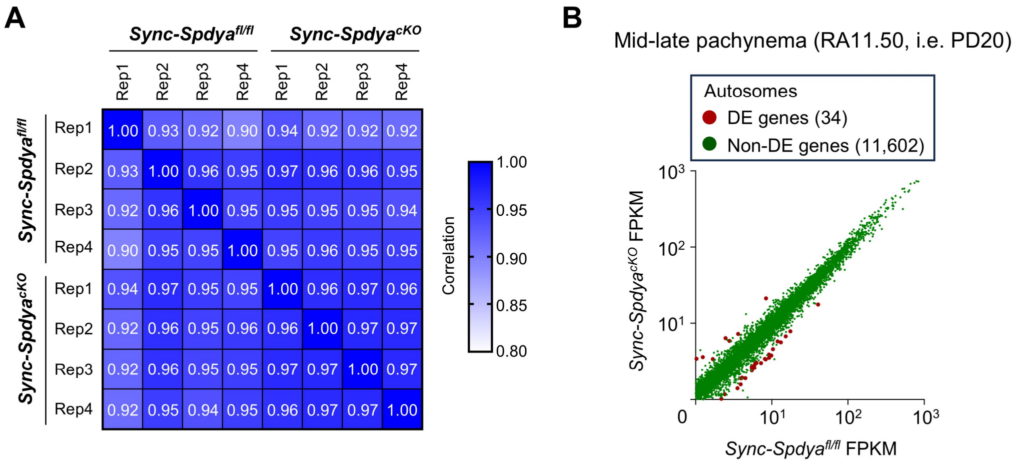


**Appendix Figure S3. Bulk RNA-seq analysis revealed similar transcriptional profiles between *Sync-Spdya*$^{fl/fl}$ and *Sync-Spdya*$^{cKO}$ mid-late pachytene testes.**

(**A**) Correlation plots of RNA-seq data from *Sync-Spdya*$^{fl/fl}$ and *Sync-Spdya*$^{cKO}$ testes collected at RA11.50 (n = 4 mice per genotype). The correlation coefficients were calculated based on reads counts from all genes in both groups.

(**B**) Differentially expressed (DE) autosomal genes in *Sync-Spdya*$^{cKO}$ vs. *Sync-Spdya*$^{fl/fl}$ spermatocytes. The numbers of DE genes (red dots) and Non-DE genes (green dots) in mid-late pachytene *Sync-Spdya*$^{cKO}$ testes relative to *Sync-Spdya*$^{fl/fl}$ testes are shown. Only protein-coding genes with an average FPKM (fragments per kilobase million) > 1 in both groups were included. *Sync-Spdya*$^{cKO}$ pachytene testes shared a similar transcriptional profile with *Sync-Spdya*$^{fl/fl}$ testes.

## Appendix Figure S4

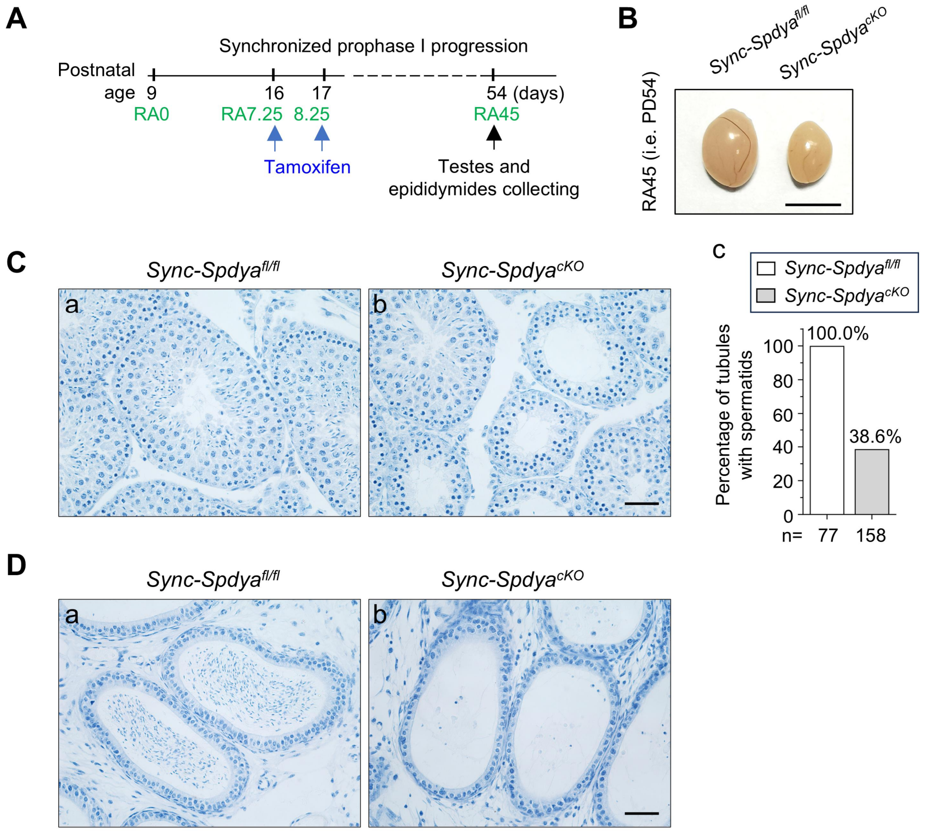


**Appendix Figure S4. Tamoxifen-induced knockout of *Spdya* in juvenile mice leads to persistent meiotic arrest and absence of spermatozoa in adulthood.**

(**A**) Synchronized mice treated with tamoxifen were allowed to grow to adulthood, and testes and epididymides were collected at RA45 (i.e., PD54, approximately 37 days after the second tamoxifen injection at RA8.25).

(**B**) The *Sync-Spdya*$^{cKO}$ testis was smaller in size than the control *Sync-Spdya*$^{fl/fl}$ testis. Scale bar, 5 mm.

(**C**) Histological analysis revealed meiotic arrest at the zygotene stage in majority of seminiferous tubules in adult *Sync-Spdya*$^{cKO}$ testes. Normal spermatogenesis was observed in some tubules, potentially derived from unaffected spermatogonia. Scale bars, 50 μm. (c) Percentages of tubules containing spermatids in the two groups. “n” represents the total number of seminiferous tubules examined.

(**D**) Compared to *Sync-Spdya*$^{fl/fl}$ mice, *Sync-Spdya*$^{cKO}$ caudal epididymides collected at RA45 lacked spermatozoa. Scale bars, 50 μm.

143 Appendix Figure S5

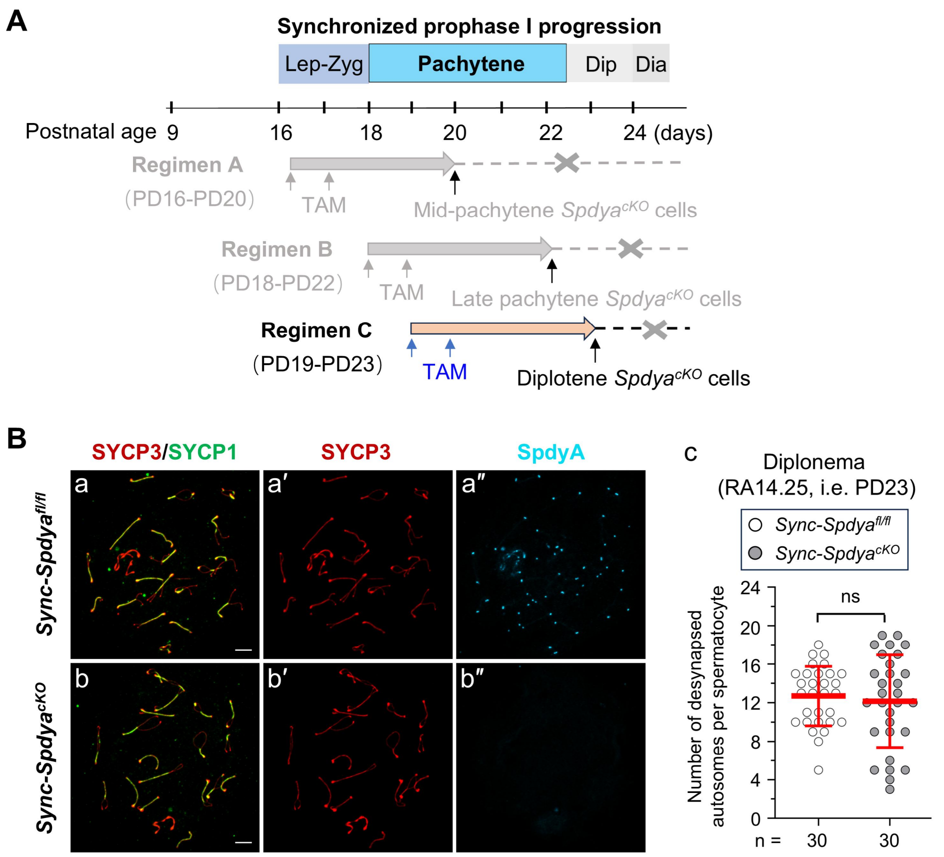


**Appendix Figure S5. Knockout of *Spdya* did not impair autosomal desynapsis.**

(**A**) Tamoxifen treatment regimen for generating diplotene *Sync-Spdya*$^{cKO}$ spermatocytes.

(**B**) Chromosome spreads of diplotene *Sync-Spdya*$^{fl/fl}$ and *Sync-Spdya*$^{cKO}$ spermatocytes immunostained for SYCP3 (red), SYCP1 (green) and SpdyA (light blue). Scale bars, 5 μm. (c) Quantification desynapsed autosomes per diplotene spermatocyte in both genotypes. Each dot represents a single nucleus. Data are presented as mean ± SD; ns, not significant (Mann–Whitney test).

## Appendix Figure S6

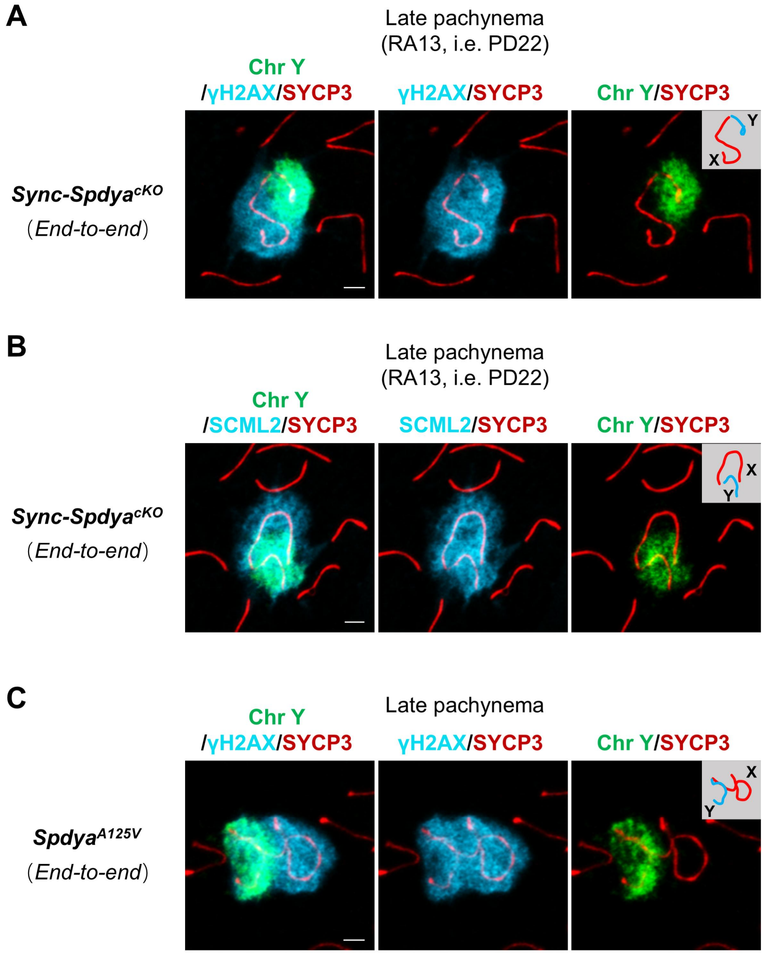


**Appendix Figure S6. Normal X-Y loop-axis organization in late pachytene *Sync-Spdya$^{cKO}$* and *Spdya$^{A125V}$* spermatocytes with end-to-end X-Y configuration.**

(**A**) Immuno-FISH analysis of late pachytene *Sync-Spdya$^{cKO}$* spermatocytes with end-to-end X-Y configuration, stained for γH2AX (light blue), Chr Y (green) and SYCP3 (red). Scale bar, 2 μm.

(**B**) Immuno-FISH analysis of late pachytene *Sync-Spdya$^{cKO}$* spermatocytes with end-to-end X-Y configuration, stained for SCML2 (light blue), Chr Y (green) and SYCP3 (red). Scale bar, 2 μm.

(**C**) Immuno-FISH analysis of late pachytene *Spdya$^{A125V}$* spermatocytes with end-to-end X-Y configuration, stained for γH2AX (light blue), Chr Y (green) and SYCP3 (red). Scale bar, 2 μm.

## Appendix Figure S7

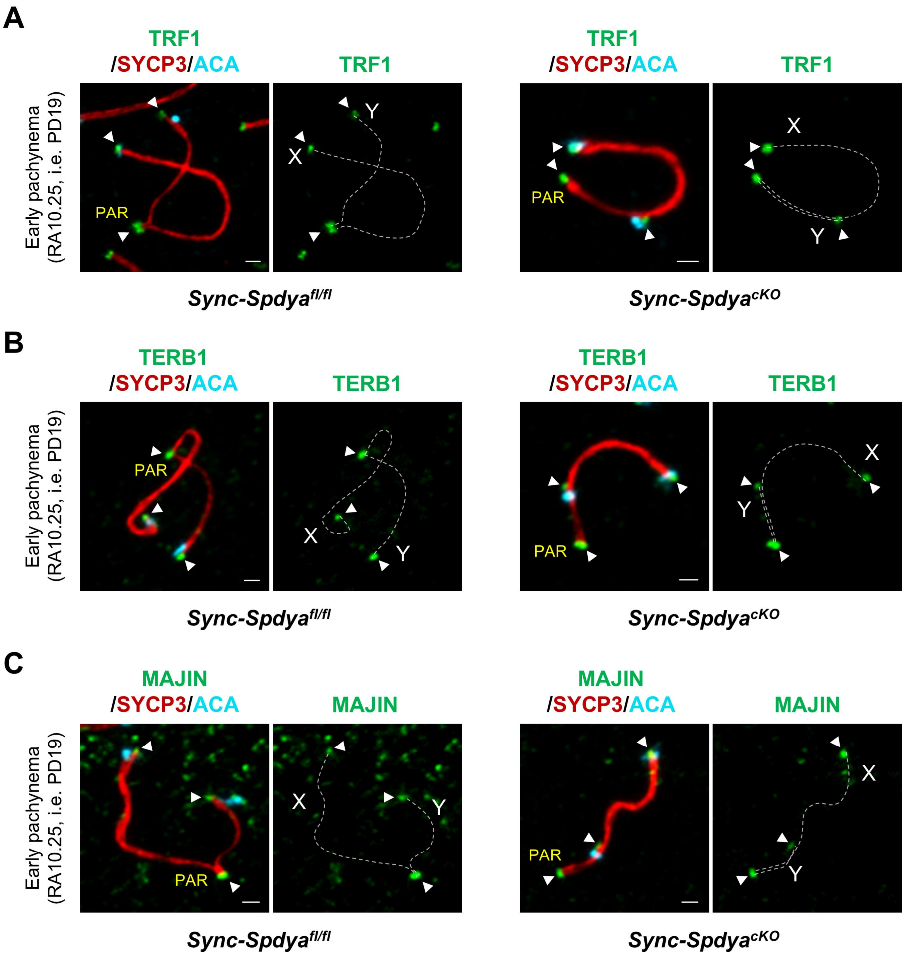


**Appendix Figure S7. Normal telomeric localization of TRF1, TERB1 and MAJIN on sex chromosome in early pachytene *Sync-Spdya*$^{cKO}$ spermatocytes.**

(**A**) Immunostaining with TRF1 (green), SYCP3 (red) and ACA (light blue). Arrowheads mark telomeres; Dashed lines outline X-Y chromosome axes. Scale bars, 1 μm.

(**B**) Immunostaining with TERB1 (green), SYCP3 (red) and ACA (light blue). Scale bars, 1 μm.

(**C**) Immunostaining with MAJIN (green), SYCP3 (red) and ACA (light blue). Scale bars, 1 μm.

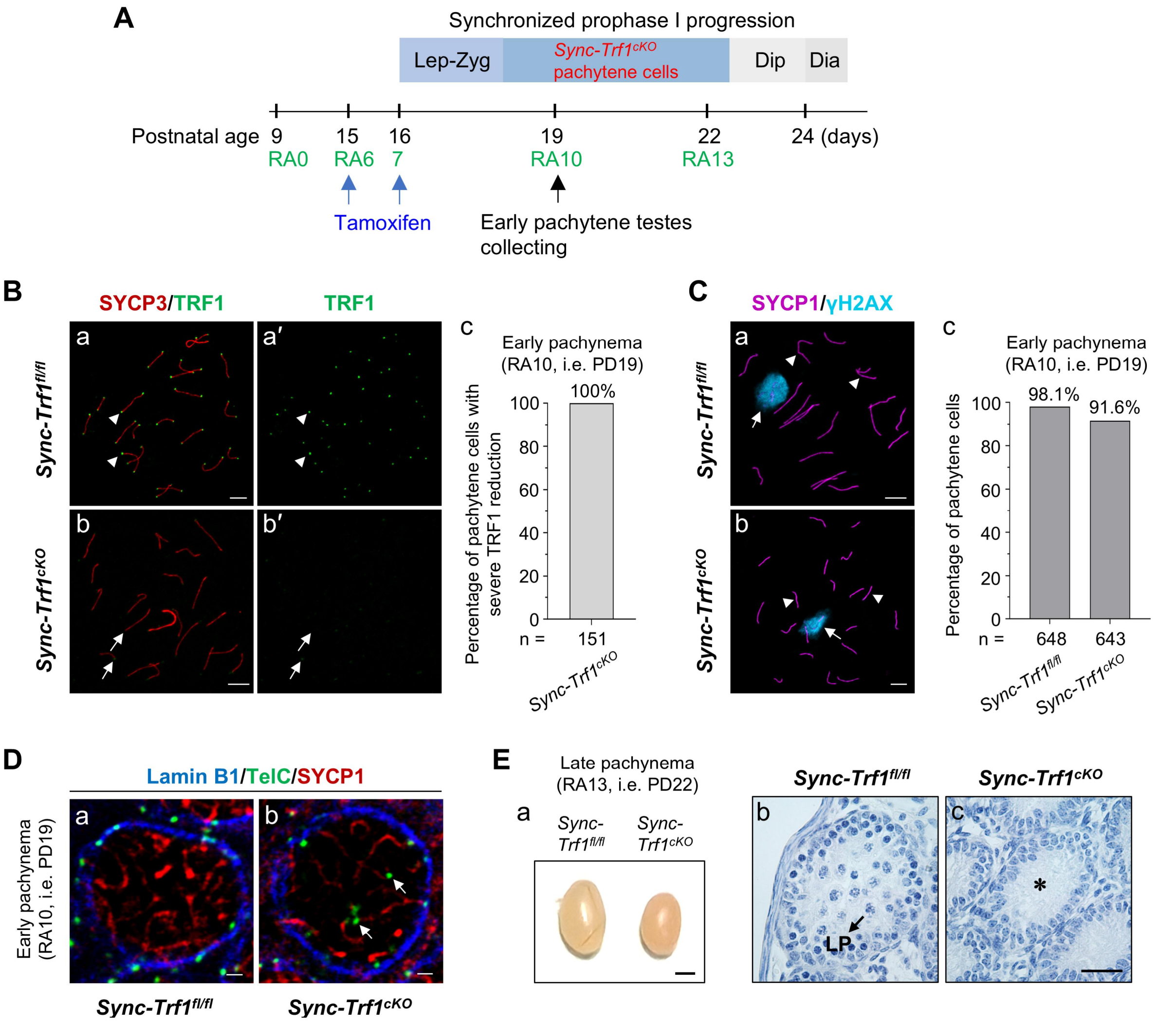


**Appendix Figure S8. Deletion of the *Trf1* gene in pachytene spermatocytes in mice.**

(**A**) Tamoxifen treatment regimen used to obtain early pachytene *Sync-Trf1$^{cKO}$* spermatocytes in synchronized *Trf1$^{fl/–}$;Ddx4-Cre$^{ERT2}$* male mice.

(**B**) Chromosome spreads of *Sync-Trf1$^{fl/fl}$* (a-a′) and *Sync-Trf1$^{cKO}$* (b-b′) spermatocytes immunostained for TRF1 (green) and SYCP3 (red). Arrowheads indicate intact TRF1 signals; arrows indicate largely disappeared TRF1 signals. Scale bars, 5 μm. (c) All analyzed pachytene cells in testes from *Trf1$^{fl/–}$;Ddx4-Cre$^{ERT2}$* mice exhibited a marked loss of TRF1 signals.

(**C**) Continuous SYCP1 signals (purple) were observed at well-synapsed autosomes (arrowheads), along with aggregated γH2AX signals (light blue) at sex chromosome areas (arrows) in *Sync-Trf1$^{cKO}$* pachytene cells. Scale bars, 5 μm. (c) Percentages of pachytene cells in *Sync-Trf1$^{fl/fl}$* and *Sync-Trf1$^{cKO}$* testes.

(**D**) Immuno-FISH analysis of testis sections stained for Lamin B1 (blue), SYCP1 (red) and TelC (green) showed impaired Tel-NE attachment in *Sync-Trf1$^{cKO}$* pachytene spermatocytes (b) at RA10. Arrows indicate telomeres displaced towards the nuclear interior, away from the NE. Scale bars, 1 μm.

(**E**) At RA13, *Sync-Trf1$^{cKO}$* testes were smaller and lacked spermatocytes. LP, late pachytene spermatocytes. Asterisk indicates loss of spermatocytes. Scale bars, 1 mm in (a); 30 μm in (b-c).

## Appendix Figure S9

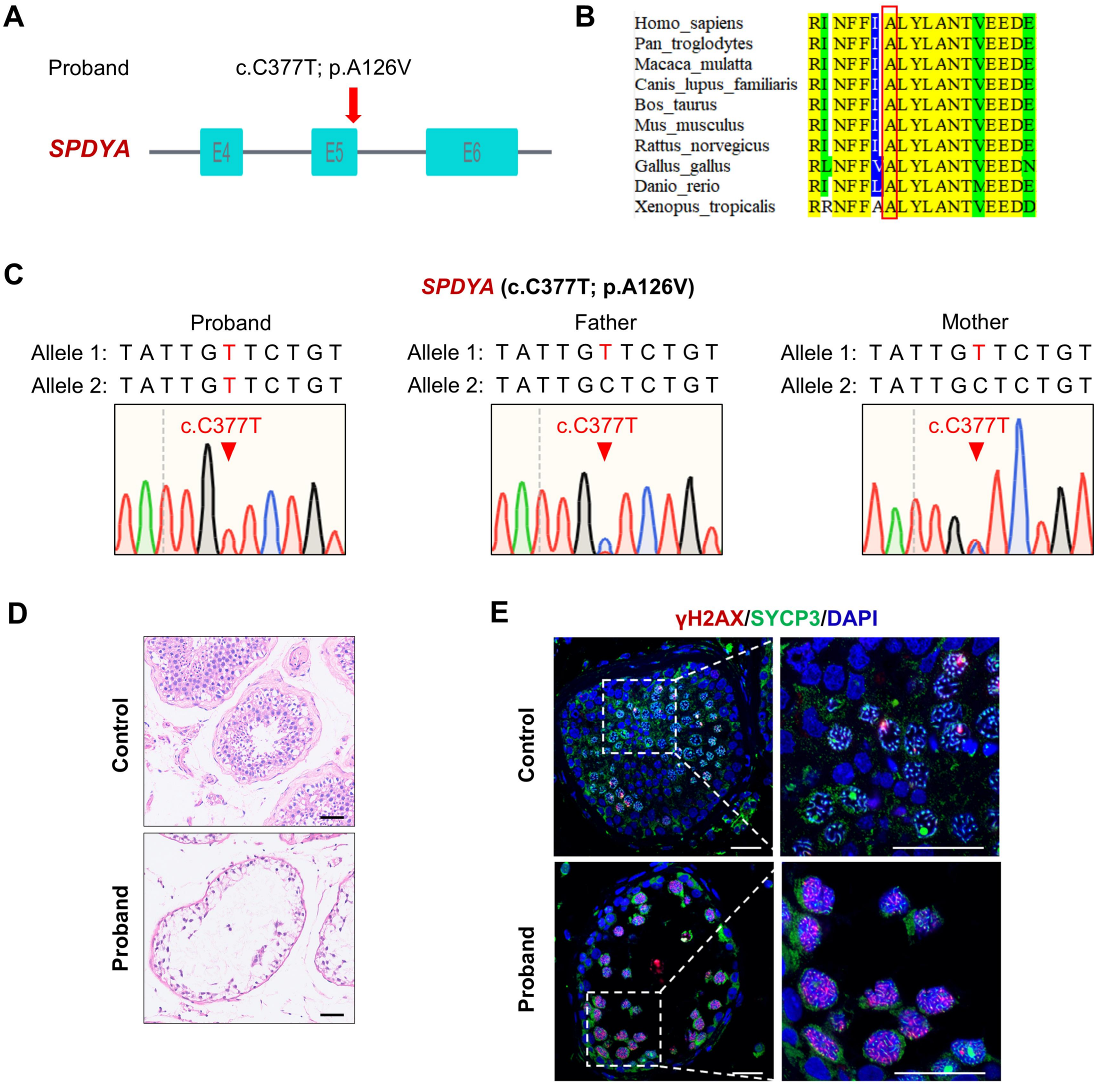


**Appendix Figure S9. Identification of a homozygous missense mutation in human *SPDYA* gene in a NOA patient.**

(**A**) Schematic representation of a homozygous missense mutation (c.C377T) in exon 5 of the *SPDYA* gene in the proband. This mutation results in an alanine (A)-to-Valine (V) substitution at the 126th residue of human SPDYA protein.

(**B**) The mutated alanine residue is highly conserved across multiple species and the substitution was predicted to be deleterious.

(**C**) Consistent with an autosomal recessive mode of inheritance, Sanger sequencing revealed that the unaffected parents were heterozygous carriers of the same *SPDYA* mutation. Arrowheads indicate the mutation sites.

(**D**) Histological analyses of testis sections from an OA patient (as control) and the proband (P21492) showed germ cell developmental arrest at the spermatocyte stage in the proband. Scale bars, 50 μm.

(**E**) IF analyses of testis sections stained with SYCP3 and γH2AX demonstrated that spermatocytes in the proband were arrested at the zygotene stage. Scale bars, 50 μm.

148 Appendix Figure S10

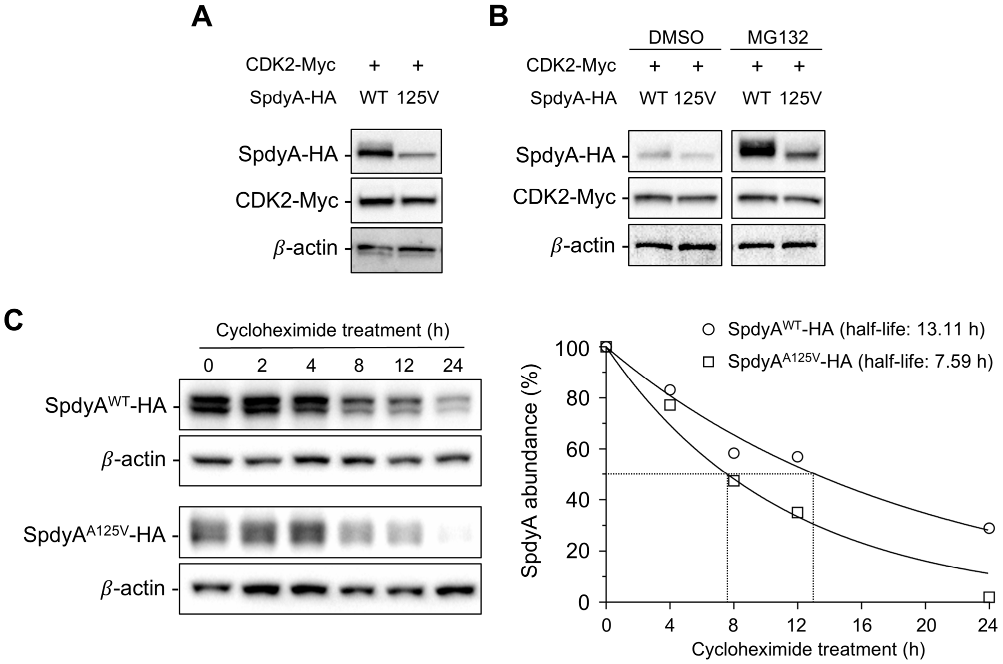


**Appendix Figure S10. The A125V substitution reduces the stability of SpdyA protein in HEK293T cells.**

(**A**) The SpdyA$^{A125V}$ mutant protein exhibited reduced stability compared to wild type SpdyA, despite the presence of CDK2. WT: wild type; 125V: A125V mutant.

(**B**) Even in the presence of the proteasome inhibitor MG132, SpdyA protein levels in the 125V group remained substantially lower than those in the WT group.

(**C**) Protein Half-lives of SpdyA$^{WT}$-HA and SpdyA$^{A125V}$-HA proteins in HEK293T cells following cycloheximide treatment. The right plot shows the protein half-life.

## Appendix Figure S11

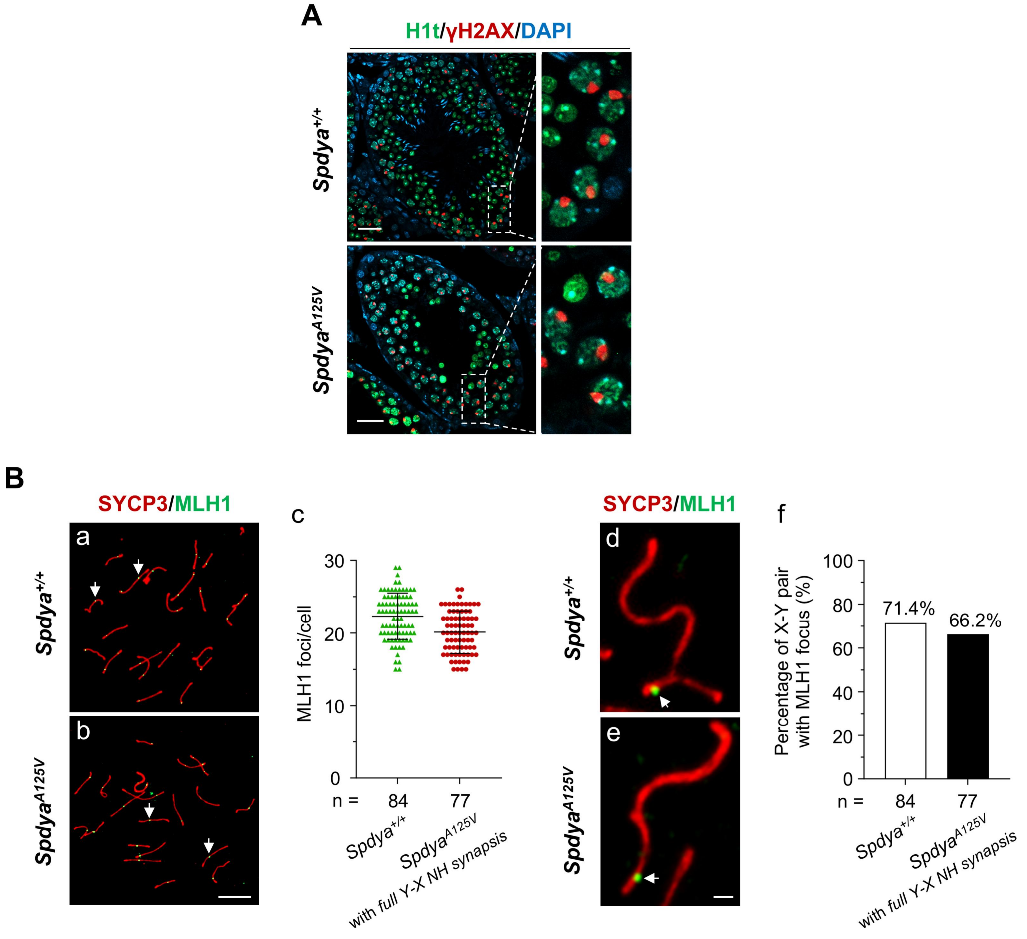


**Appendix Figure S11. Normal meiotic progression and crossover formation in *Spdya^A125V^* pachytene spermatocytes.**

(**A**) IF analyses of adult *Spdya*$^{+/+}$ and *Spdya*$^{A125V}$ testis sections immunostained for H1t (green) and γH2AX (red). Nuclei were counterstained with DAPI. Scale bars, 30 μm.

(**B**) Surface spreads of *Spdya*$^{+/+}$ and *Spdya*$^{A125V}$ spermatocytes immunostained for SYCP3 (red) and MLH1 (green; arrows). Scale bars, 5 μm. (c) Scatter plot of MLH1 foci numbers per cell in *Spdya*$^{+/+}$ (green triangles) and *Spdya*$^{A125V}$ (red dots) pachytene spermatocytes. Data are presented as mean ± SD. (d-e) Representative images of sex chromosomes with a single MLH1 focus (arrows) in *Spdya*$^{+/+}$ and *Spdya*$^{A125V}$ pachytene cells. Scale bars, 1 μm. (f) The percentage of XY pairs with an MLH1 focus in the PAR was comparable between *Spdya*$^{+/+}$ and *Spdya*$^{A125V}$ pachytene spermatocytes with *full Y-X NH synapsis*.

# Appendix Figure S12

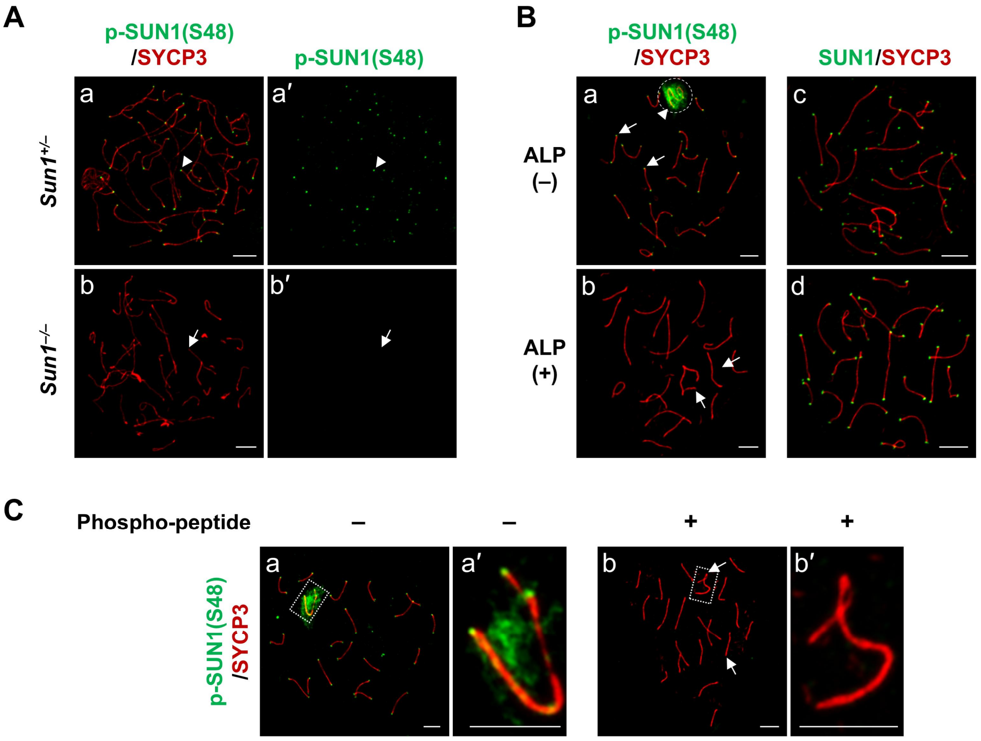


**Appendix Figure S12. The p-SUN1(S48) were specifically localized at telomeres in prophase I mouse spermatocytes.**

(**A**) In *Sun1*$^{+/–}$ zygotene spermatocytes, p-SUN1(S48) signals were clearly detected at telomeres (a-a′, arrowheads), whereas in *Sun1*$^{–/–}$ zygotene-like cells, p-SUN1(S48) signals were absent from telomeres (b-b′, arrows). Scale bars, 5 μm.

(**B**). In wild type pachytene cells, p-SUN1(S48) signals at telomeres (a, arrows) were abolished following alkaline phosphatase (ALP) treatment (b, arrows), while total SUN1 signals remained unaffected (d). Arrowhead with dashed circle in (a) marks a non-specific p-SUN1(S48) signal at sex chromosome area in pachytene cells. Scale bars, 5 μm.

(**C**) Preabsorption of the antibody with the phosphorylated antigenic peptide abolished p-SUN1(S48) signals at telomeres in wild-type pachytene cells (b, arrows), confirming signal specificity. Scale bars, 5 μm.

## Appendix Figure S13

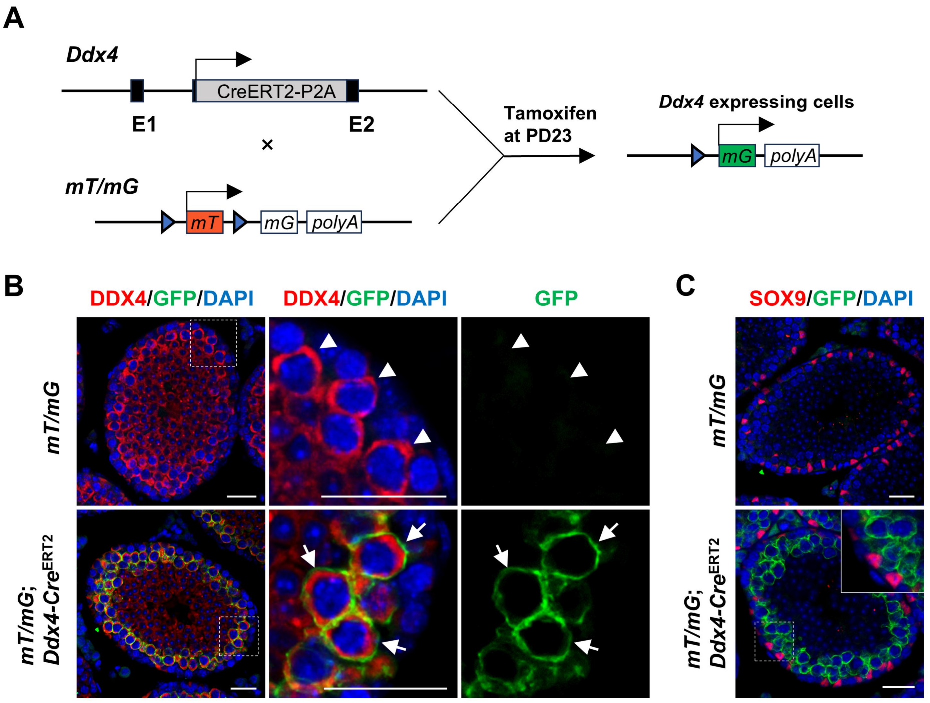


**Appendix Figure S13. Functional validation of *Ddx4-Cre$^{ERT2}$* knockin mice using *mT/mG* reporter mice.**

(**A**) Schematic illustration of tamoxifen-induced labeling of germ cells in *mT/mG;Ddx4-Cre$^{ERT2}$* mice. A single *intraperitoneal (i.p.)* injection of tamoxifen (20 mg/kg body weight) was administered to PD23 *mT/mG* and *mT/mG;Ddx4-Cre$^{ERT2}$* mice. Testes were collected and analyzed 2 days after tamoxifen treatment.

(**B**) IF analyses of testis sections immunostained for GFP and the germ cell specific marker DDX4 showed EGFP fluorescence specifically in DDX4-positive cells (arrows), indicating germ cell-specific and tamoxifen-inducible Cre recombinase activity in *Ddx4-Cre$^{ERT2}$* mice. As a control, no Cre activity was observed in DDX4-positive cells of *mT/mG* mice treated with the same tamoxifen regimen (arrowheads). Scale bars, 30 μm.

(**C**) IF analyses of testis sections immunostained for GFP and the somatic marker SOX9 (sertoli cells) showed no evidence of Cre activity in somatic cells following tamoxifen induction. Scale bars, 30 μm.

**Appendix Table S1.** Information of the primers used for genotyping, Sanger sequencing and quantitative PCR.

| Information | Name | Sequence (5'-3') |
|---|---|---|
| For genotyping of the *Spdya* conditional knockout mice | Spdya-flox/cKO-F4 | TATTAGGTATAGCCTCACCTCCAGC |
| | Spdya-flox-R4 | AGATCCTAATGAGACCCTCTGATCT |
| | Spdya-cKO-R5 | TTCTTACTTTTTCACAATGTTCC |
| For genotyping of the *Ddx4-Cre*$^{ERT2}$ knock-in mice | Ddx4CreERT2-F4 | TAAAGATCACCCTTAGGCAAGACG |
| | Ddx4CreERT2-F5 | CGCATTCCTTGCAAAAGTATTACA |
| | Ddx4CreERT2-R5 | ATGCAGATAAACACTGAAACAGGC |
| For genotyping of the *Trf1* conditional knockout mice | Trf1-flox/cKO-F1 | GACATGGTAGTACATAACTGTGTTCC |
| | Trf1-flox-R1 | CTGGCCATCTTGGAACTCTTCCTAT |
| | Trf1-cKO-R3 | AAGGTTTACAGCACAACTCTAACC |
| For genotyping of the *Stra8-EGFPCre* knock-in mice | Stra8EGFPCre-F1 | GATGGATTTCCGTCTCTGGTGTAG |
| | Stra8EGFPCre-F2 | GTCAGAGAAGGTTGTATCGAACTGG |
| | Stra8EGFPCre-R1 | CCCATTTAATCTCCTCCTTCTCCG |
| For Sanger sequencing of the *Spdya*$^{A125V}$ mutant mice | Spdya-WT/125V-F1 | AACGATTCAAGTGGATTAACTCTGG |
| | Spdya-WT/125V-R1 | TGGCATGTAATGTAAGGCCAATGA |

**Appendix Table S2.** Clinical and semen characteristics in the proband with the bi-allelic *SPDYA* variant with A126V missense mutation.

| | **Subject** | |
|---|---|---|
| | P21492 | Reference |
| **Age (years)** | 30 | / |
| **Testicular volume (left) (ml)** | 12 | 12~15 |
| **Testicular volume (right) (ml)** | 12 | 12~15 |
| **FSH (IU/L)** | 3.11 | 1.27~19.26 |
| **LH (IU/L)** | 2.15 | 1.24~8.62 |
| **T (μg/L)** | 2.93 | 1.75~7.81 |
| **Karyotype** | 46, XY | 46, XY |
| **Y Chromosome Microdeletions** | N | N |
| **Semen parameters** | | |
| **Semen volume (ml)** | 2.50 | ≥1.5 |
| **Sperm concentration ($10^6$/ml)** | 0 | ≥15 |
| **PR (%)** | 0 | ≥32 |
| **NP (%)** | 0 | / |
| **IM (%)** | 0 | / |
| **Centrifuged spermatozoa number (/ejaculate)** | 0 | / |

Abbreviations: FSH, follicle-stimulating hormone; LH, luteinizing hormone; T, testosterone; PR, progressive; NP, non-progressive; IM, immobility; N, normal phenotype.

**Appendix Table S3.** List of antibodies.

| Antibody | Host | Dilution | Company | Cat # |
|---|---|---|---|---|
| SYCP3 | Mouse | IF: 1:200 | Abcam | ab97672 |
| SYCP3 | Chicken | IF: 1:200 | This paper | NA |
| SYCP1 | Guinea pig | IF: 1:200<br>IHC: 1:200 | This paper | NA |
| SYCE1 | Rabbit | IF: 1:200 | Proteintech | 11063-1-AP |
| TEX12 | Rabbit | IF: 1:200 | Proteintech | 17068-1-AP |
| SPDYA | Rat | IF: 1:200 | This paper | NA |
| $\gamma$H2AX | Mouse | IF: 1:2000<br>IHC: 1:1000 | Millipore | 05-636 |
| $\gamma$H2AX | Rabbit | IF: 1:400<br>IHC: 1:400 | Cell Signaling Technology | 9718 |
| SCML2 | Rabbit | IF: 1:200 | Kindly provided by Prof. Mengcheng Luo (Luo *et al*, 2015) | NA |
| FK2 | Mouse | IF: 1:100 | Millipore | 04-263 |
| ACA | Human | IF: 1:200<br>IHC: 1:100 | Antibodiesinc | 15-234 |
| H1t | Rat | IHC: 1:200 | This paper | NA |
| MLH1 | Mouse | IF: 1:50 | BD Pharmingen$^{TM}$ | 51-1327GR |
| TRF1 | Mouse | IF: 1:100<br>IHC: 1:100 | Abcam | ab10579 |
| TERB1 | Rabbit | IF: 1:200 | This paper | NA |
| MAJIN | Rabbit | IF: 1:100 | This paper | NA |
| SUN1 | Rabbit | IF: 1:400 | This paper | NA |
| KASH5 | Rabbit | IF: 1:200 | This paper | NA |
| ANKRD31 | Rabbit | IF: 1:200 | This paper | NA |
| p-SUN1(S48) | Rabbit | IF: 1:200<br>WB: 1:1000 | This paper | NA |
| HORMAD1 | Rabbit | IHC: 1:200 | Proteintech | 13917-1-AP |
| Lamin B1 | Rabbit | IHC: 1:200 | Proteintech | 12987-1-AP |
| Cleaved PARP (Asp214) | Rabbit | IHC: 1:200 | Cell Signaling Technology | 94885 |
| macroH2A1 | Rabbit | IF: 1:100 | Sigma-Aldrich | ABE215 |
| GFP | Chicken | IHC: 1:200 | Abcam | Ab13970 |
| DDX4 | Mouse | IHC: 1:200 | Abcam | Ab27591 |
| SOX9 | Rabbit | IHC: 1:200 | Millipore | AB5535 |
| CDK2 | Rabbit | WB: 1:1000 | Abcam | ab32147 |

| | | | | |
|---|---|---|---|---|
| ACTB | Mouse | WB: 1:10000 | Proteintech | 66009-1-IG |
| HA | Rabbit | WB: 1:1000 | Cell Signaling Technology | 3724 |
| FLAG | Rabbit | WB: 1:1000 | Cell Signaling Technology | 14793 |
| MYC | Mouse | WB: 1:1000 | Proteintech | 60003-2-IG |
| HIS | Rabbit | WB: 1:1000 | Proteintech | 10001-0-AP |

## Appendix Reference

Al Sorkhy M, Craig R, Market B, Ard R, Porter LA (2009) The cyclin-dependent kinase activator, Spy1A, is targeted for degradation by the ubiquitin ligase NEDD4. *The Journal of biological chemistry* 284: 2617-2627

Chen Y, Wang Y, Chen J, Zuo W, Fan Y, Huang S, Liu Y, Chen G, Li Q, Li J *et al* (2021) The SUN1-SPDYA interaction plays an essential role in meiosis prophase I. *Nature communications* 12: 3176

Luo M, Zhou J, Leu NA, Abreu CM, Wang J, Anguera MC, de Rooij DG, Jasin M, Wang PJ (2015) Polycomb protein SCML2 associates with USP7 and counteracts histone H2A ubiquitination in the XY chromatin during male meiosis. *PLoS genetics* 11: e1004954

Schneider-Poetsch T, Ju J, Eyler DE, Dang Y, Bhat S, Merrick WC, Green R, Shen B, Liu JO (2010) Inhibition of eukaryotic translation elongation by cycloheximide and lactimidomycin. *Nat Chem Biol* 6: 209-217

Wang L, Tu Z, Liu C, Liu H, Kaldis P, Chen Z, Li W (2018) Dual roles of TRF1 in tethering telomeres to the nuclear envelope and protecting them from fusion during meiosis. *Cell death and differentiation* 25: 1174-1188
