# Supplementary figures and images for: Speedy A governs non-homologous XY chromosome desynapsis as a unique prerequisite for XY loop-axis organization
Source: EMBO J. 2025 Aug 18;44(19):5509–36. doi: 10.1038/s44318-025-00528-8 (PMC12488978; doi:10.1038/s44318-025-00528-8)

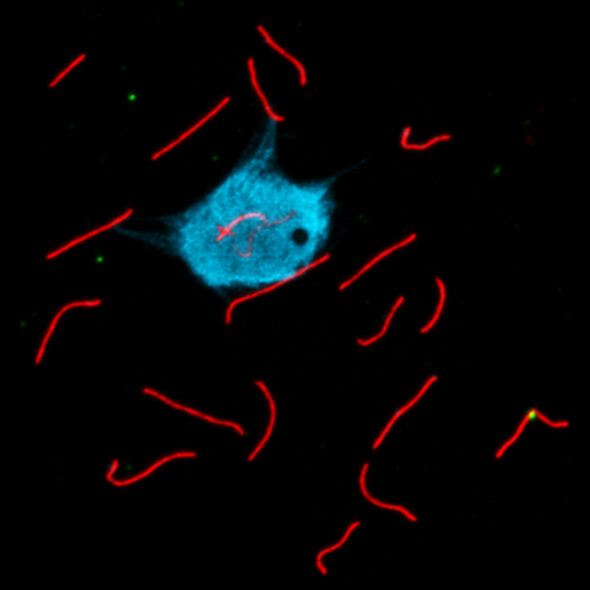

Supplement: Supplementary file 5 — Source data Fig. 1 [file 44318_2025_528_MOESM5_ESM.zip › Figure 1/1B/Spdyacko-SpdyA&SYCP1&gammaH2AX.tif]

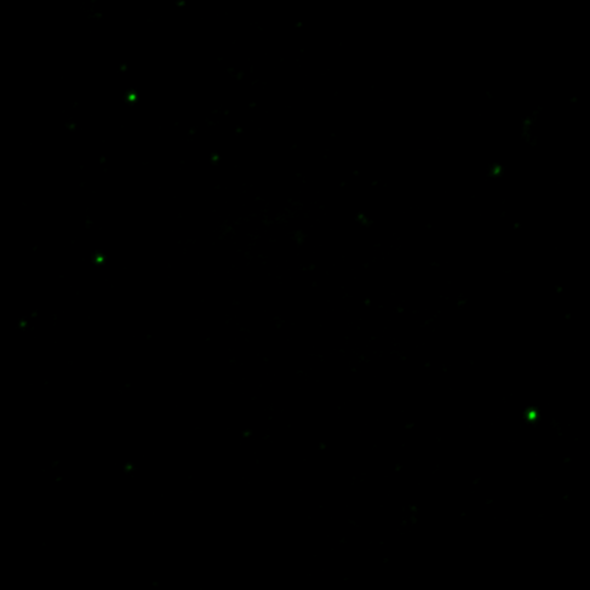

Supplement: Supplementary file 5 — Source data Fig. 1 [file 44318_2025_528_MOESM5_ESM.zip › Figure 1/1B/Spdyacko-SpdyA.tif]

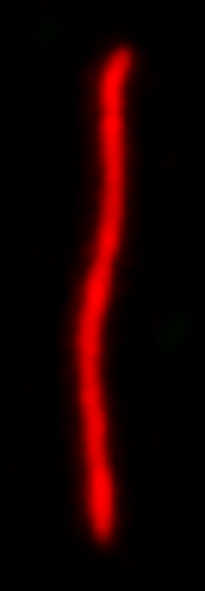

Supplement: Supplementary file 5 — Source data Fig. 1 [file 44318_2025_528_MOESM5_ESM.zip › Figure 1/1B/Spdyacko-SYCP1&SpdyA-Aut.tif]

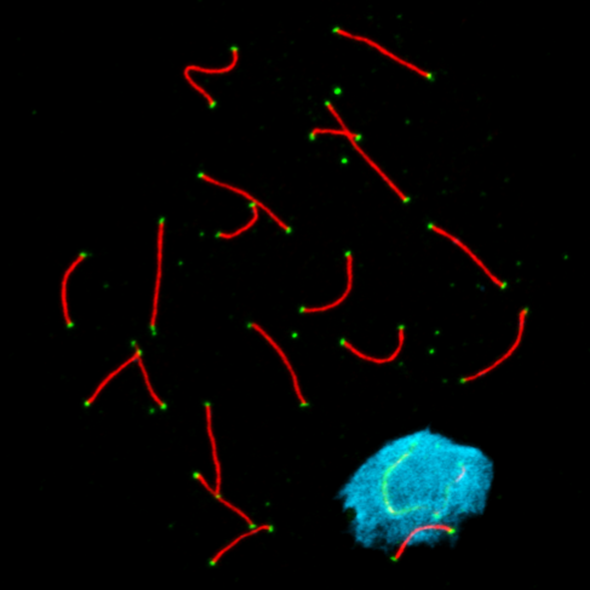

Supplement: Supplementary file 5 — Source data Fig. 1 [file 44318_2025_528_MOESM5_ESM.zip › Figure 1/1B/Spdyaflfl-SpdyA&SYCP1&gammaH2AX.tif]

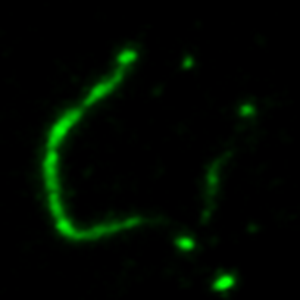

Supplement: Supplementary file 5 — Source data Fig. 1 [file 44318_2025_528_MOESM5_ESM.zip › Figure 1/1B/Spdyaflfl-SpdyA-XY.tif]

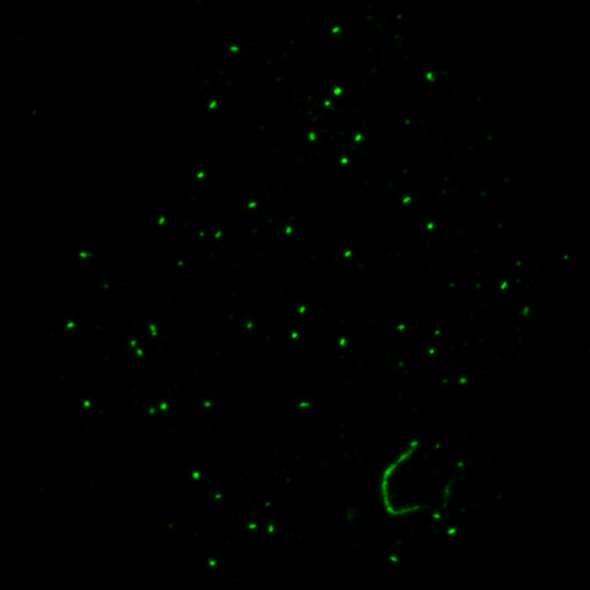

Supplement: Supplementary file 5 — Source data Fig. 1 [file 44318_2025_528_MOESM5_ESM.zip › Figure 1/1B/Spdyaflfl-SpdyA.tif]

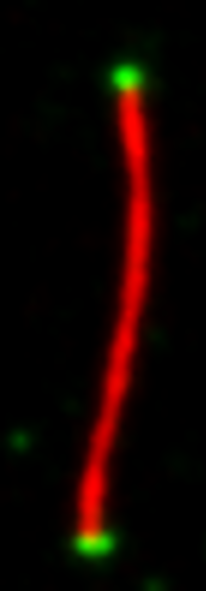

Supplement: Supplementary file 5 — Source data Fig. 1 [file 44318_2025_528_MOESM5_ESM.zip › Figure 1/1B/Spdyaflfl-SYCP1&SpdyA-Aut.tif]

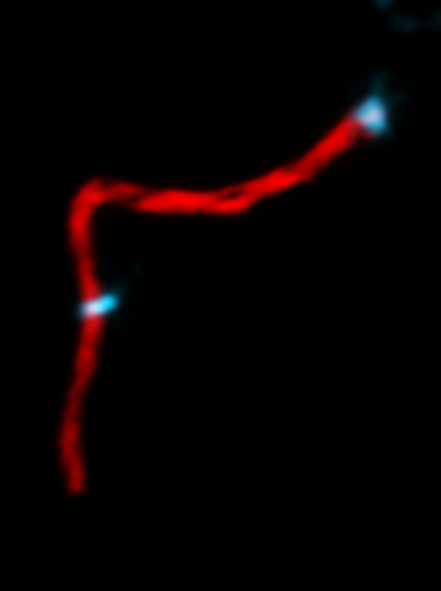

Supplement: Supplementary file 5 — Source data Fig. 1 [file 44318_2025_528_MOESM5_ESM.zip › Figure 1/1C/Spdyacko-SYCP3&ACA-XY.tif]

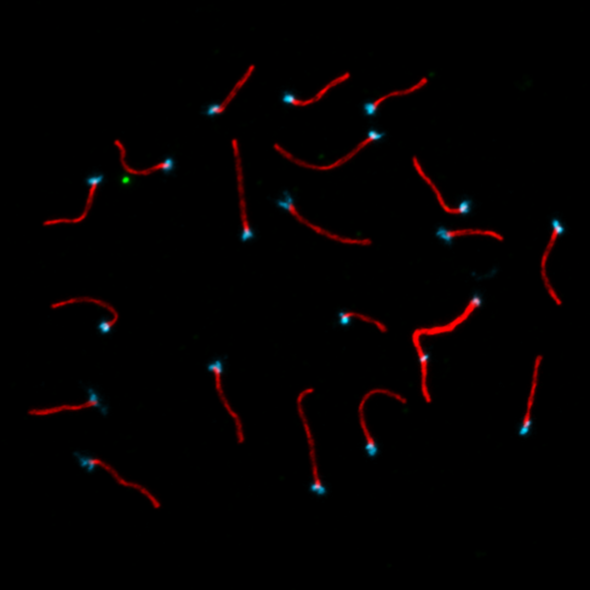

Supplement: Supplementary file 5 — Source data Fig. 1 [file 44318_2025_528_MOESM5_ESM.zip › Figure 1/1C/Spdyacko-SYCP3&SpdyA&ACA.tif]

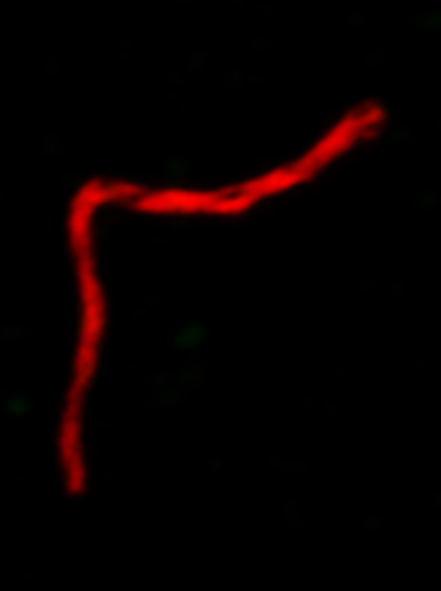

Supplement: Supplementary file 5 — Source data Fig. 1 [file 44318_2025_528_MOESM5_ESM.zip › Figure 1/1C/Spdyacko-SYCP3&SpdyA-XY.tif]

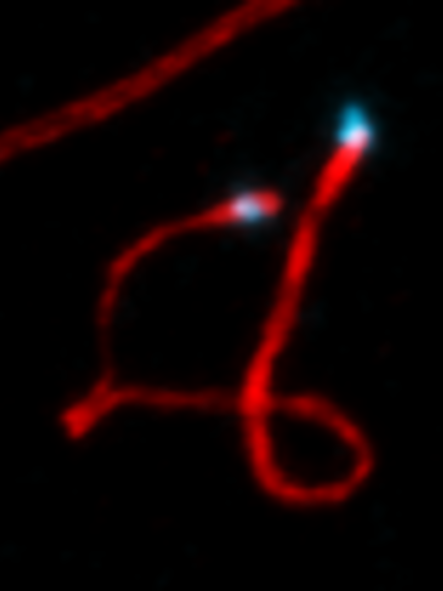

Supplement: Supplementary file 5 — Source data Fig. 1 [file 44318_2025_528_MOESM5_ESM.zip › Figure 1/1C/Spdyaflfl-SYCP3&ACA-XY.tif]

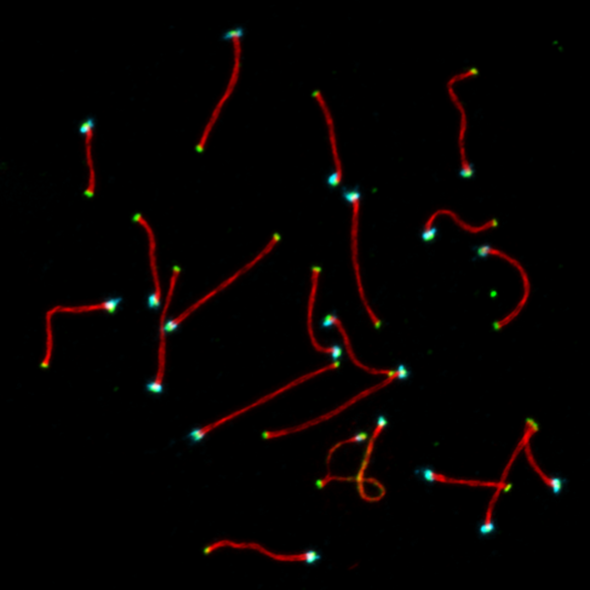

Supplement: Supplementary file 5 — Source data Fig. 1 [file 44318_2025_528_MOESM5_ESM.zip › Figure 1/1C/Spdyaflfl-SYCP3&SpdyA&ACA.tif]

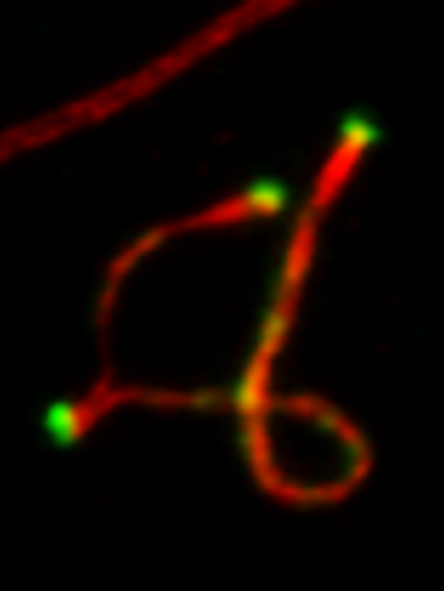

Supplement: Supplementary file 5 — Source data Fig. 1 [file 44318_2025_528_MOESM5_ESM.zip › Figure 1/1C/Spdyaflfl-SYCP3&SpdyA-XY.tif]

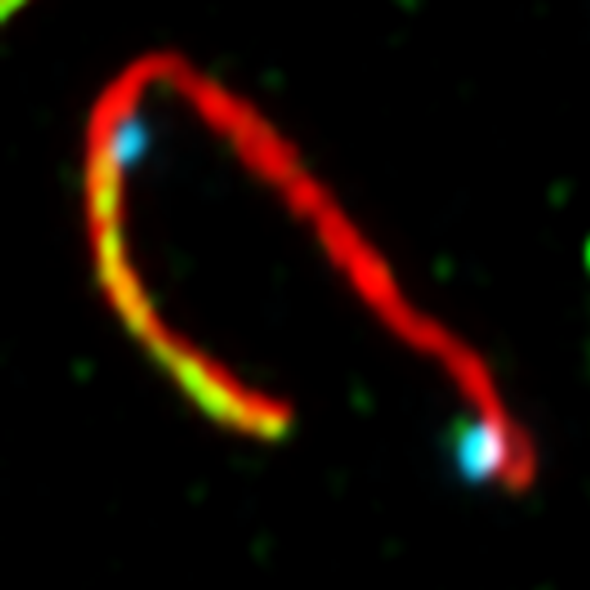

Supplement: Supplementary file 5 — Source data Fig. 1 [file 44318_2025_528_MOESM5_ESM.zip › Figure 1/1D/Spdyacko-XY-SYCE1&SYCP3&ACA.tif]

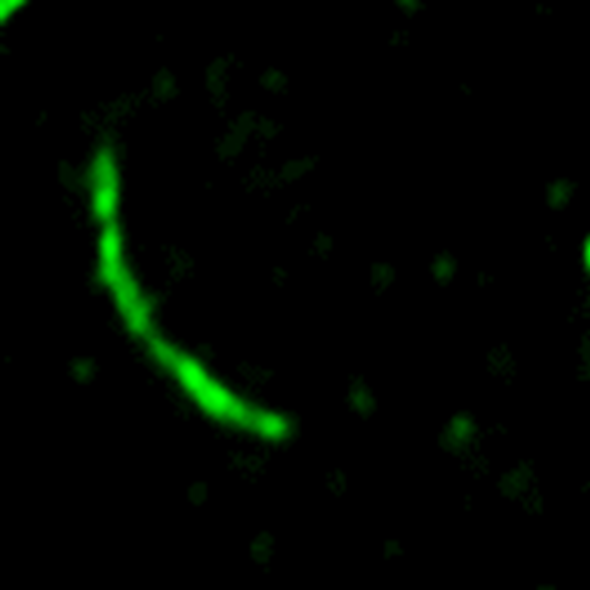

Supplement: Supplementary file 5 — Source data Fig. 1 [file 44318_2025_528_MOESM5_ESM.zip › Figure 1/1D/Spdyacko-XY-SYCE1.tif]

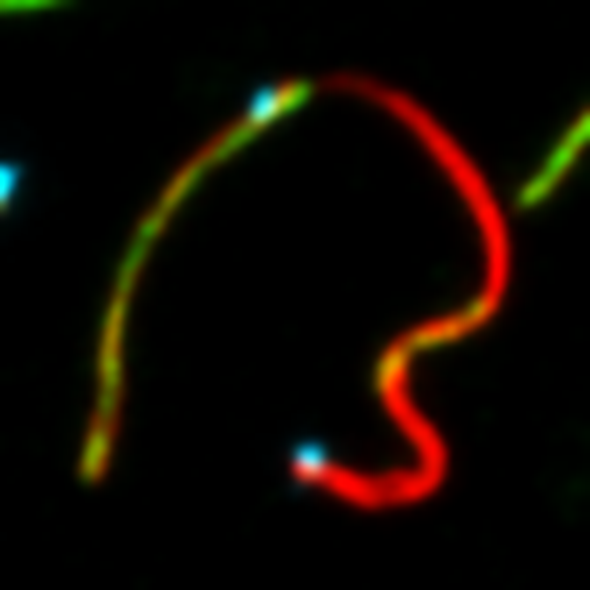

Supplement: Supplementary file 5 — Source data Fig. 1 [file 44318_2025_528_MOESM5_ESM.zip › Figure 1/1D/Spdyacko-XY-SYCP1&SYCP3&ACA.tif]

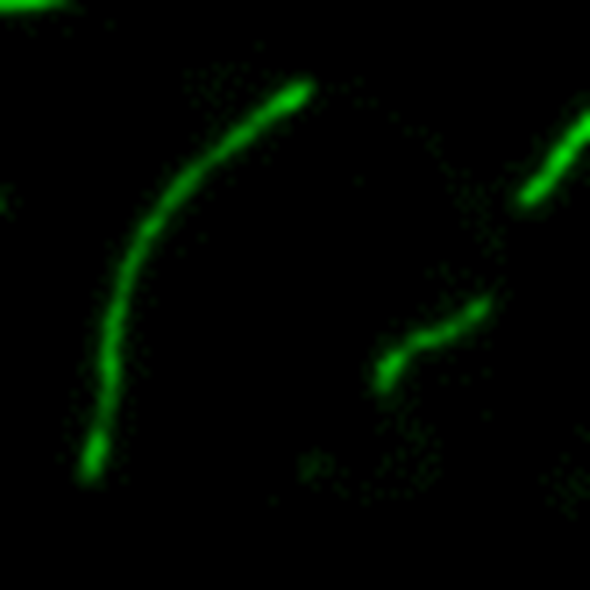

Supplement: Supplementary file 5 — Source data Fig. 1 [file 44318_2025_528_MOESM5_ESM.zip › Figure 1/1D/Spdyacko-XY-SYCP1.tif]

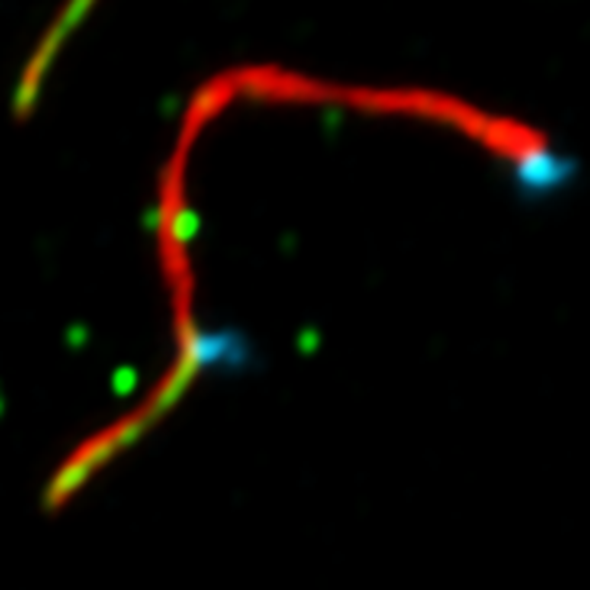

Supplement: Supplementary file 5 — Source data Fig. 1 [file 44318_2025_528_MOESM5_ESM.zip › Figure 1/1D/Spdyacko-XY-TEX12&SYCP3&ACA.tif]

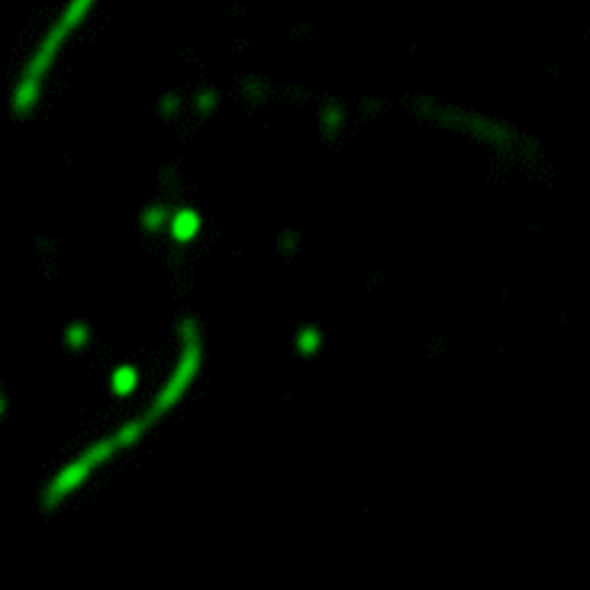

Supplement: Supplementary file 5 — Source data Fig. 1 [file 44318_2025_528_MOESM5_ESM.zip › Figure 1/1D/Spdyacko-XY-TEX12.tif]

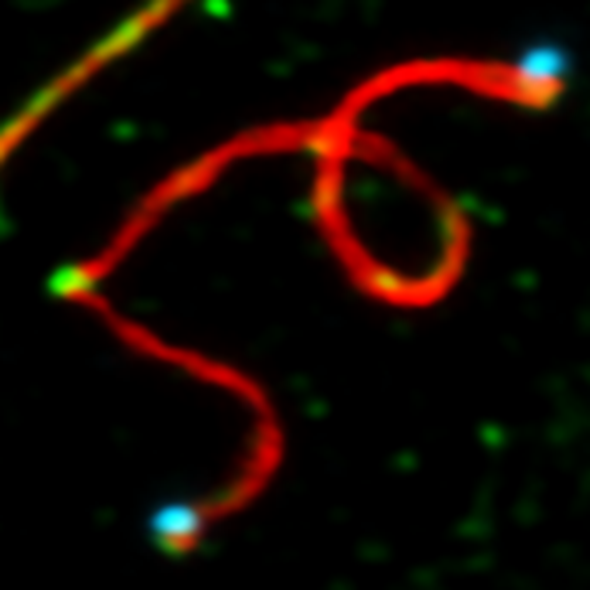

Supplement: Supplementary file 5 — Source data Fig. 1 [file 44318_2025_528_MOESM5_ESM.zip › Figure 1/1D/Spdyaflfl-XY-SYCE1&SYCP3&ACA.tif]

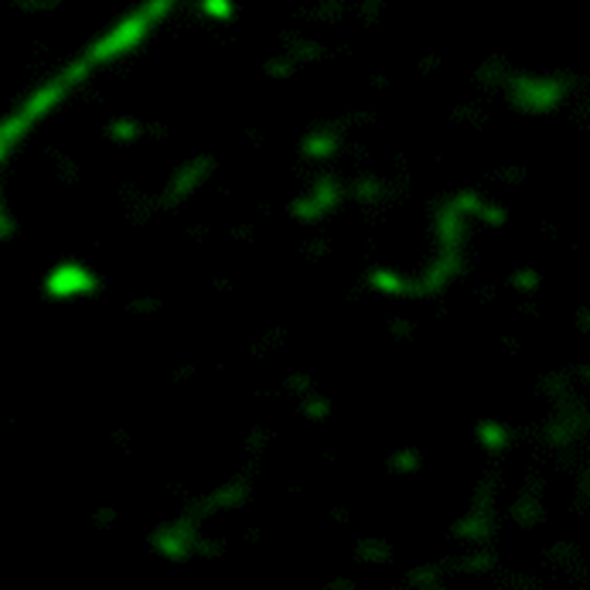

Supplement: Supplementary file 5 — Source data Fig. 1 [file 44318_2025_528_MOESM5_ESM.zip › Figure 1/1D/Spdyaflfl-XY-SYCE1.tif]

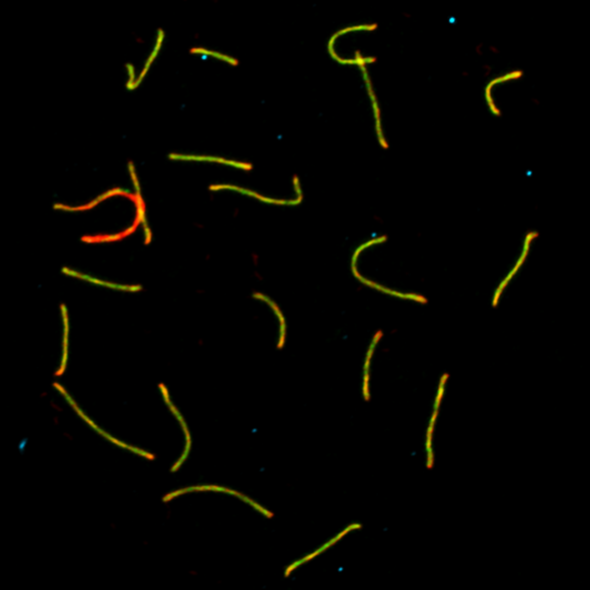

Supplement: Supplementary file 6 — Source data Fig. 2 [file 44318_2025_528_MOESM6_ESM.zip › Figure 2/2B/SyncSpdyacko-SYCP3&SYCP1&SpdyA.tif]

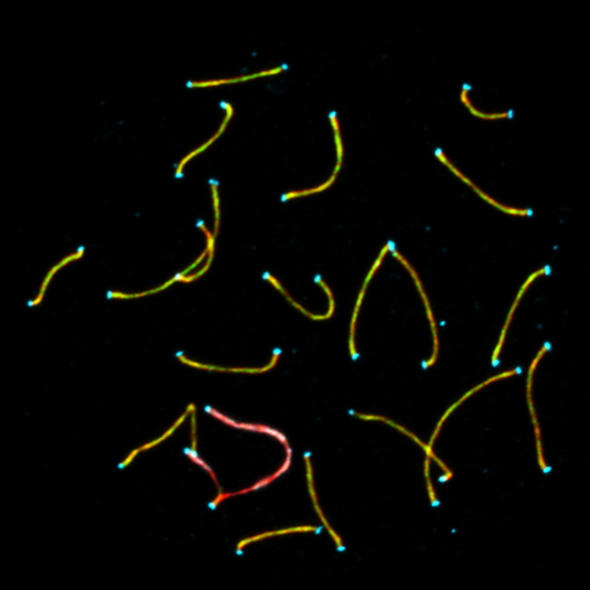

Supplement: Supplementary file 6 — Source data Fig. 2 [file 44318_2025_528_MOESM6_ESM.zip › Figure 2/2B/SyncSpdyaflfl-SYCP3&SYCP1&SpdyA.tif]

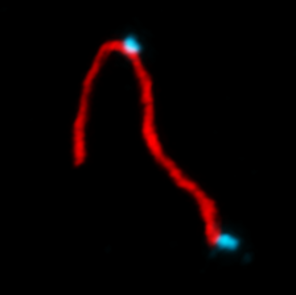

Supplement: Supplementary file 6 — Source data Fig. 2 [file 44318_2025_528_MOESM6_ESM.zip › Figure 2/2C/SyncSpdyacko-XY-SYCP3&ACA.tif]

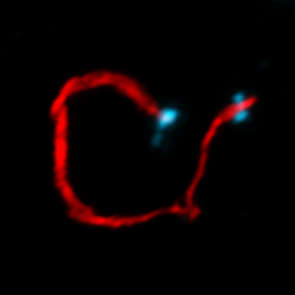

Supplement: Supplementary file 6 — Source data Fig. 2 [file 44318_2025_528_MOESM6_ESM.zip › Figure 2/2C/SyncSpdyaflfl-XY-SYCP3&ACA.tif]

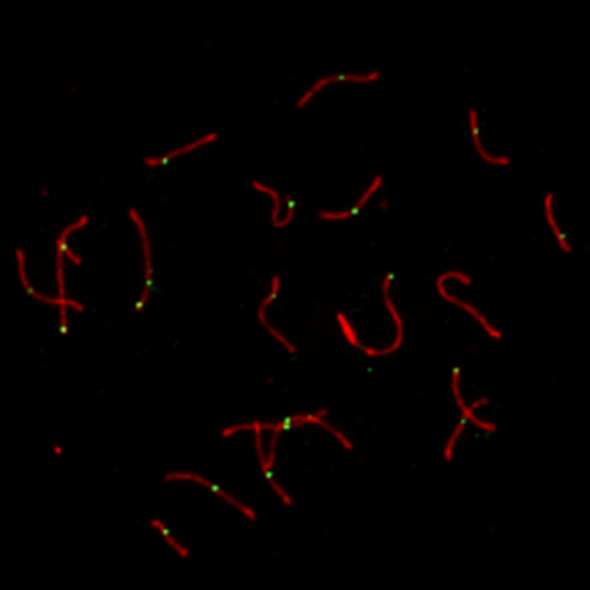

Supplement: Supplementary file 6 — Source data Fig. 2 [file 44318_2025_528_MOESM6_ESM.zip › Figure 2/2D/SyncSpdyacko-SYCP3&MLH1.tif]

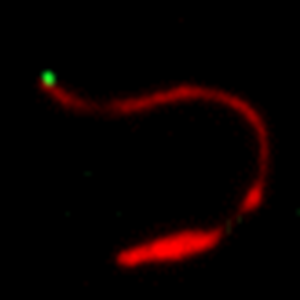

Supplement: Supplementary file 6 — Source data Fig. 2 [file 44318_2025_528_MOESM6_ESM.zip › Figure 2/2D/SyncSpdyacko-XY-SYCP3&MLH1.tif]

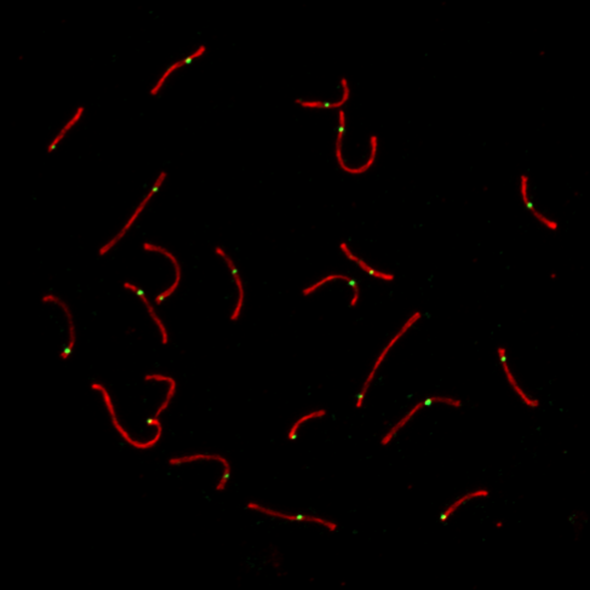

Supplement: Supplementary file 6 — Source data Fig. 2 [file 44318_2025_528_MOESM6_ESM.zip › Figure 2/2D/SyncSpdyaflfl-SYCP3&MLH1.tif]

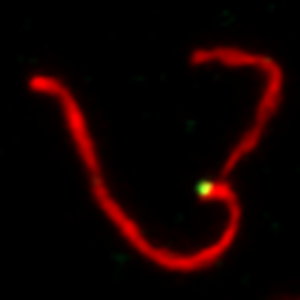

Supplement: Supplementary file 6 — Source data Fig. 2 [file 44318_2025_528_MOESM6_ESM.zip › Figure 2/2D/SyncSpdyaflfl-XY-SYCP3&MLH1.tif]

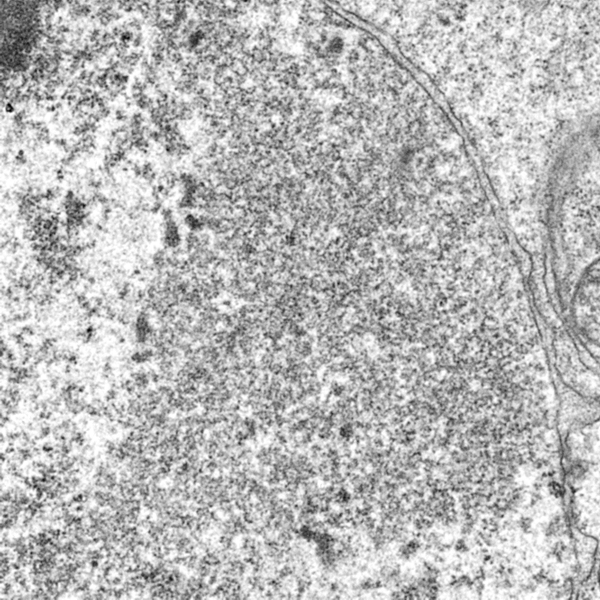

Supplement: Supplementary file 6 — Source data Fig. 2 [file 44318_2025_528_MOESM6_ESM.zip › Figure 2/2F/SyncSpdyacko-SEM-SB.tif]

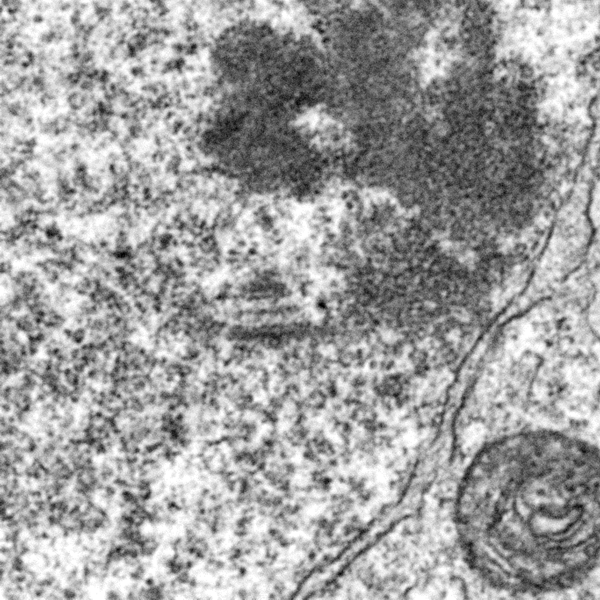

Supplement: Supplementary file 6 — Source data Fig. 2 [file 44318_2025_528_MOESM6_ESM.zip › Figure 2/2F/SyncSpdyacko-SEM-SC.tif]

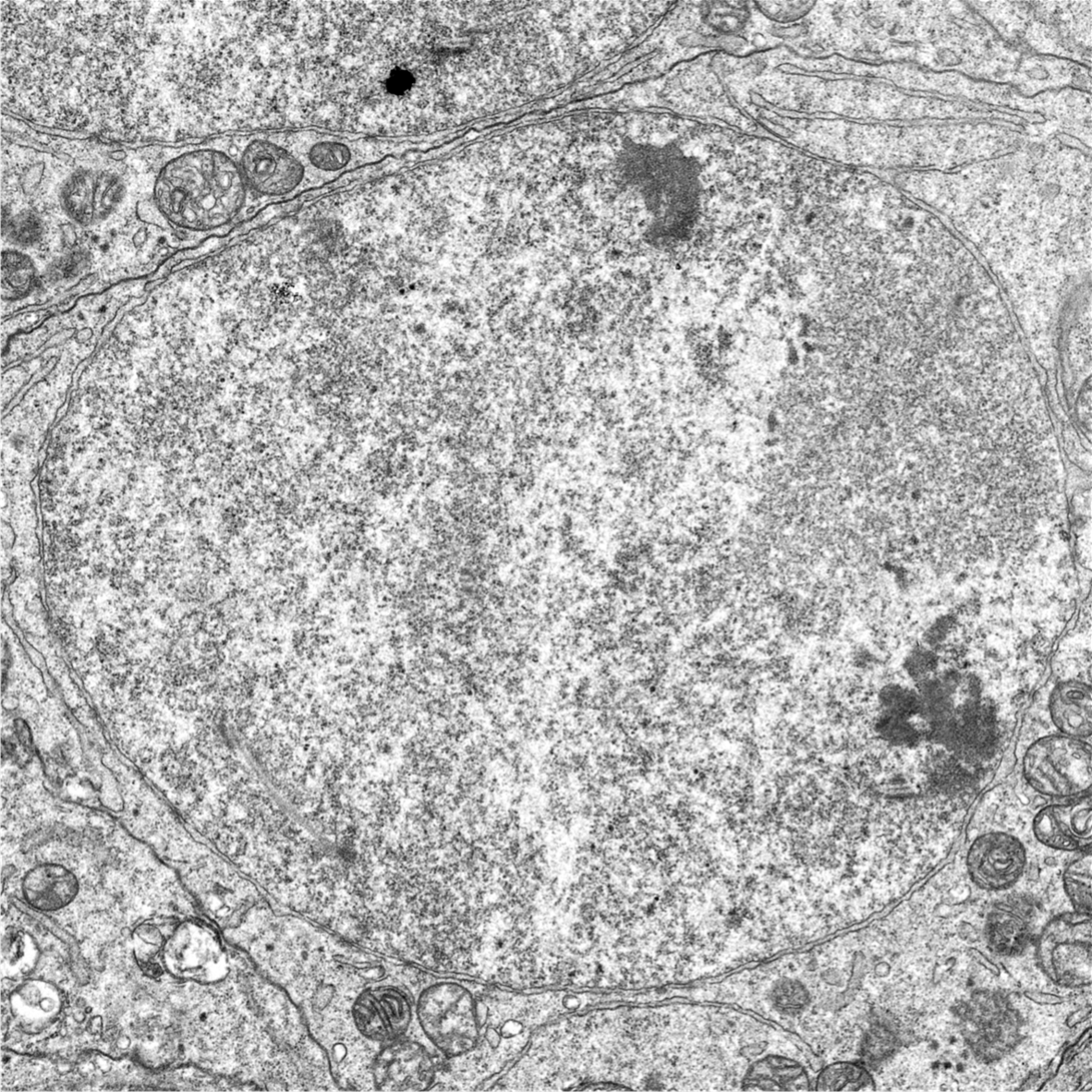

Supplement: Supplementary file 6 — Source data Fig. 2 [file 44318_2025_528_MOESM6_ESM.zip › Figure 2/2F/SyncSpdyacko-SEM.tif]

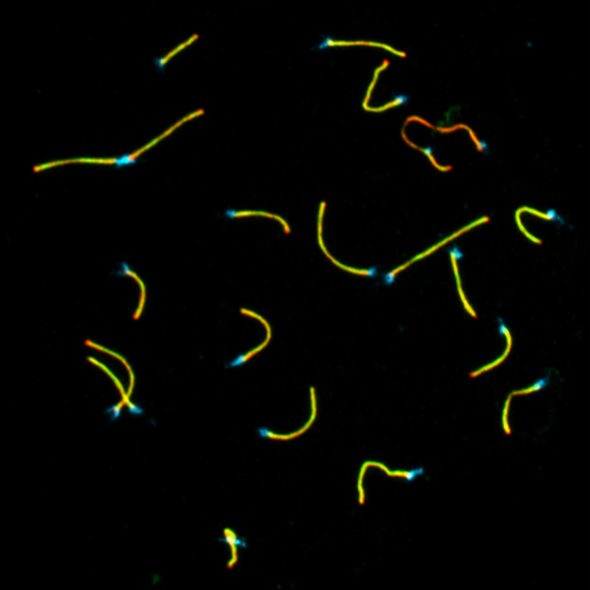

Supplement: Supplementary file 7 — Source data Fig. 3 [file 44318_2025_528_MOESM7_ESM.zip › Figure 3/3C/SyncSpdyacko-SYCP3&SYCP1&ACA.tif]

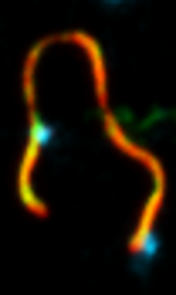

Supplement: Supplementary file 7 — Source data Fig. 3 [file 44318_2025_528_MOESM7_ESM.zip › Figure 3/3C/SyncSpdyacko-XY-SYCP3&SYCP1&ACA.tif]

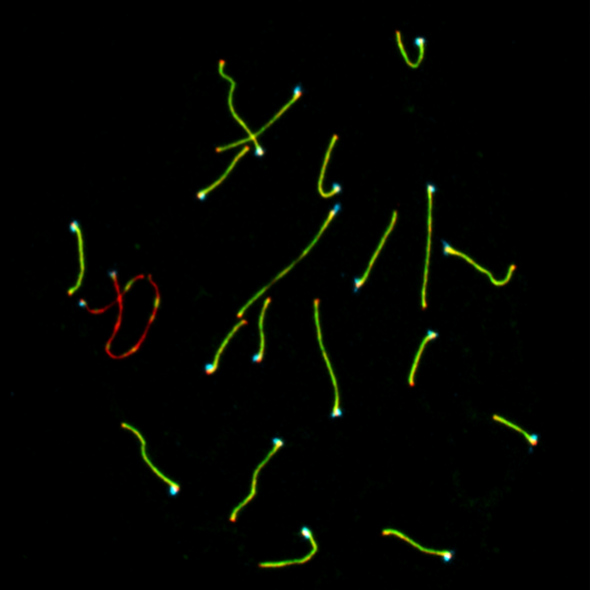

Supplement: Supplementary file 7 — Source data Fig. 3 [file 44318_2025_528_MOESM7_ESM.zip › Figure 3/3C/SyncSpdyaflfl-SYCP3&SYCP1&ACA.tif]

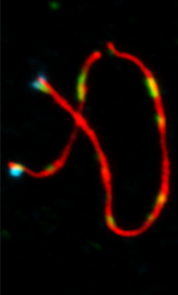

Supplement: Supplementary file 7 — Source data Fig. 3 [file 44318_2025_528_MOESM7_ESM.zip › Figure 3/3C/SyncSpdyaflfl-XY-SYCP3&SYCP1&ACA.tif]

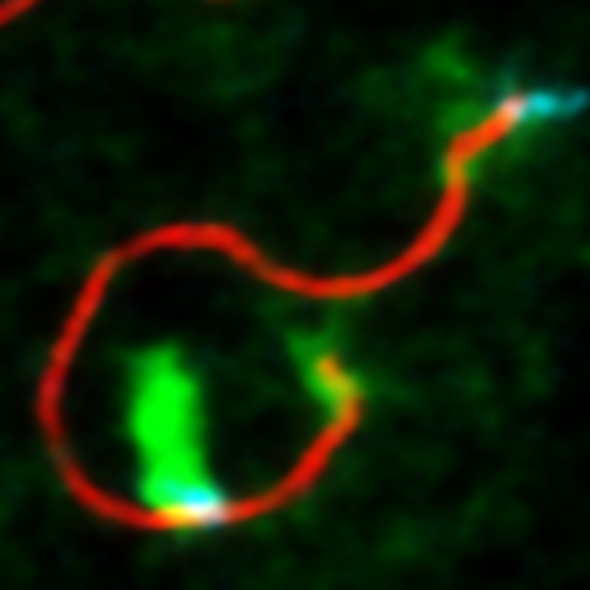

Supplement: Supplementary file 7 — Source data Fig. 3 [file 44318_2025_528_MOESM7_ESM.zip › Figure 3/3D/SyncSpdyacko-XY-macroH2A1&SYCP3&ACA-LP.tif]

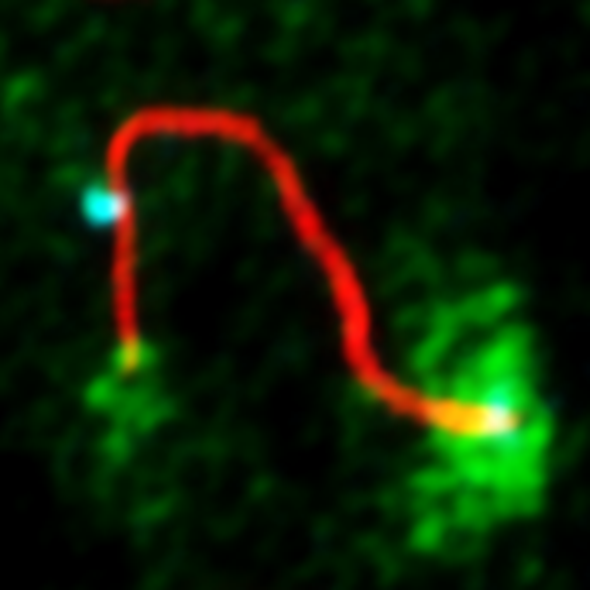

Supplement: Supplementary file 7 — Source data Fig. 3 [file 44318_2025_528_MOESM7_ESM.zip › Figure 3/3D/SyncSpdyacko-XY-macroH2A1&SYCP3&ACA-MLP.tif]

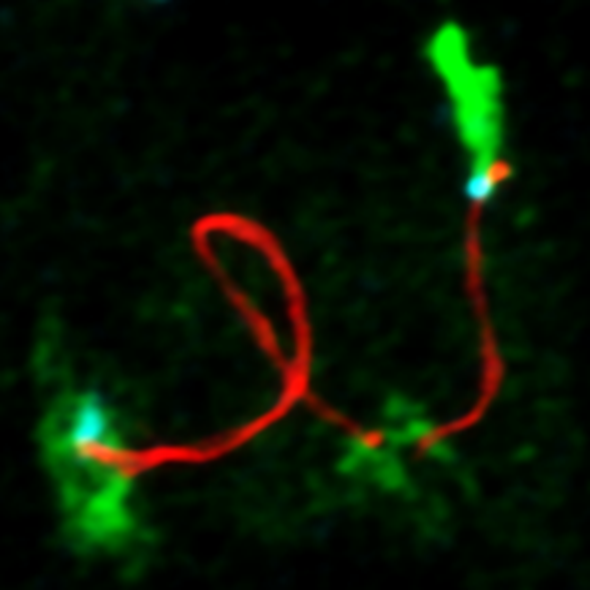

Supplement: Supplementary file 7 — Source data Fig. 3 [file 44318_2025_528_MOESM7_ESM.zip › Figure 3/3D/SyncSpdyaflfl-XY-macroH2A1&SYCP3&ACA-LP.tif]

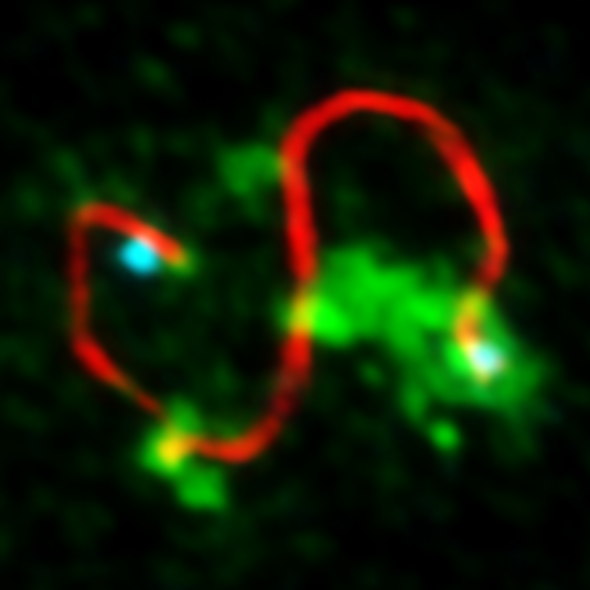

Supplement: Supplementary file 7 — Source data Fig. 3 [file 44318_2025_528_MOESM7_ESM.zip › Figure 3/3D/SyncSpdyaflfl-XY-macroH2A1&SYCP3&ACA-MLP.tif]

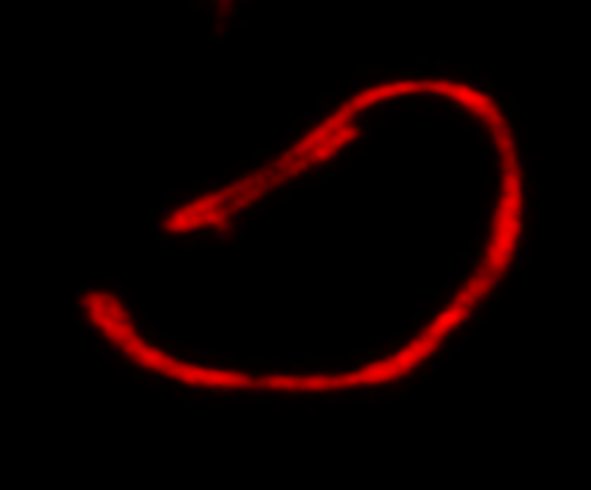

Supplement: Supplementary file 8 — Source data Fig. 4 [file 44318_2025_528_MOESM8_ESM.zip › Figure 4/4A/SyncSpdyacko-SYCP3.tif]

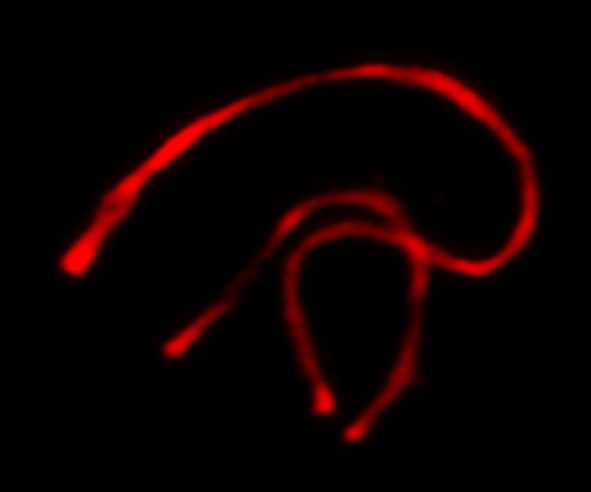

Supplement: Supplementary file 8 — Source data Fig. 4 [file 44318_2025_528_MOESM8_ESM.zip › Figure 4/4A/SyncSpdyaflfl-SYCP3.tif]

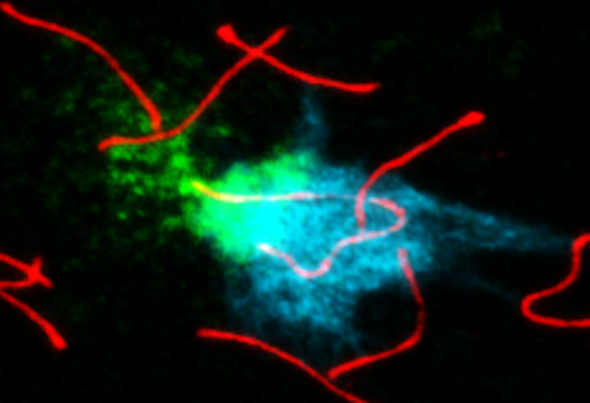

Supplement: Supplementary file 8 — Source data Fig. 4 [file 44318_2025_528_MOESM8_ESM.zip › Figure 4/4B/SyncSpdyacko-ChrY&gammaH2AX&SYCP3.tif]

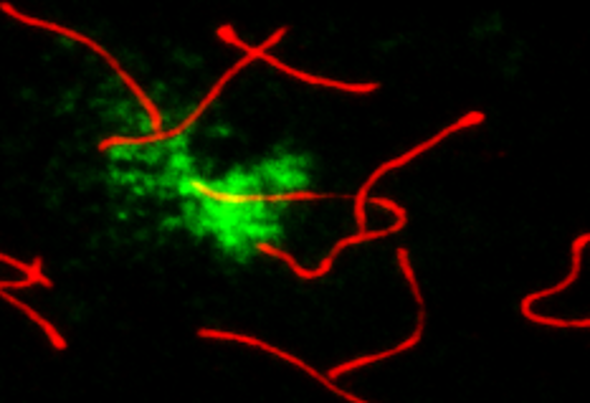

Supplement: Supplementary file 8 — Source data Fig. 4 [file 44318_2025_528_MOESM8_ESM.zip › Figure 4/4B/SyncSpdyacko-ChrY&SYCP3.tif]

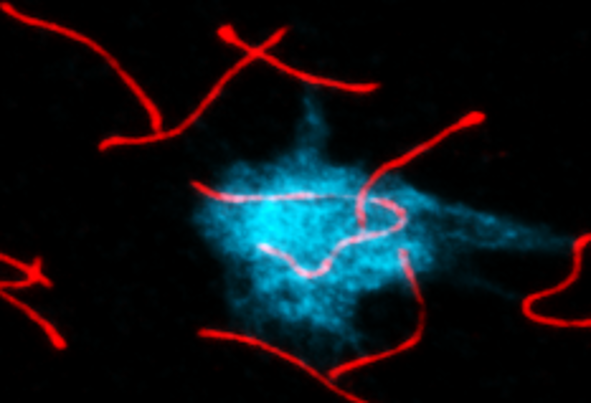

Supplement: Supplementary file 8 — Source data Fig. 4 [file 44318_2025_528_MOESM8_ESM.zip › Figure 4/4B/SyncSpdyacko-gammaH2AX&SYCP3.tif]

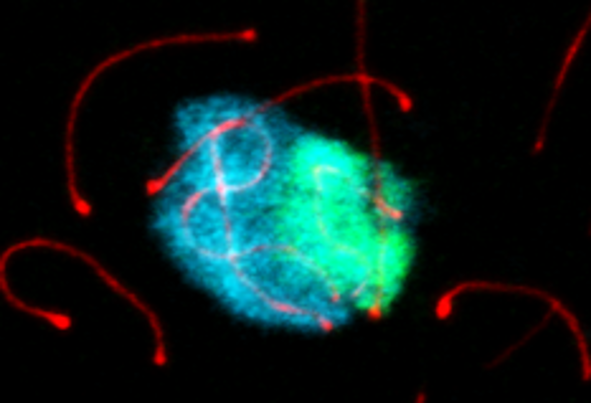

Supplement: Supplementary file 8 — Source data Fig. 4 [file 44318_2025_528_MOESM8_ESM.zip › Figure 4/4B/SyncSpdyaflfl-ChrY&gammaH2AX&SYCP3.tif]

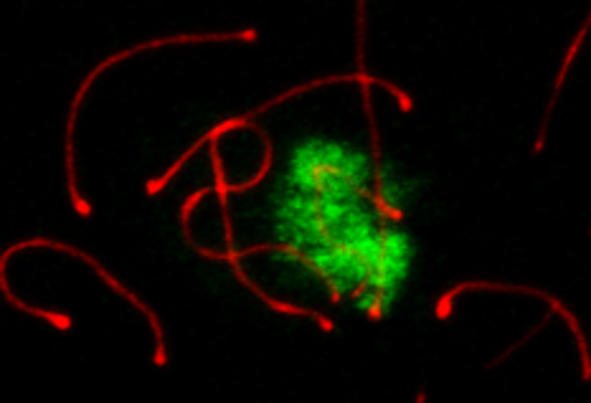

Supplement: Supplementary file 8 — Source data Fig. 4 [file 44318_2025_528_MOESM8_ESM.zip › Figure 4/4B/SyncSpdyaflfl-ChrY&SYCP3.tif]

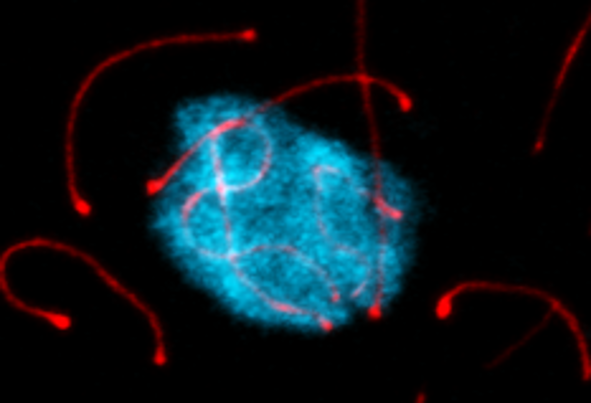

Supplement: Supplementary file 8 — Source data Fig. 4 [file 44318_2025_528_MOESM8_ESM.zip › Figure 4/4B/SyncSpdyaflfl-gammaH2AX&SYCP3.tif]

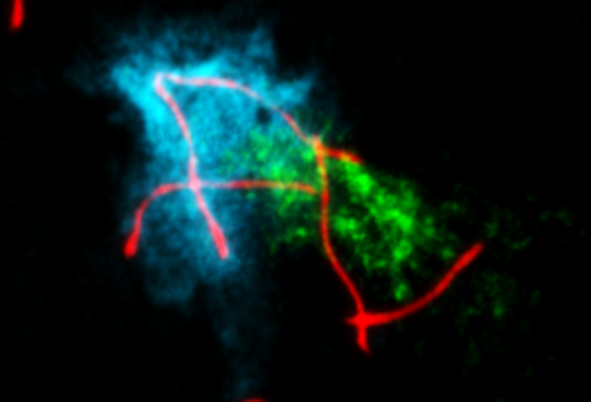

Supplement: Supplementary file 8 — Source data Fig. 4 [file 44318_2025_528_MOESM8_ESM.zip › Figure 4/4C/SyncSpdyacko-ChrY&SCML2&SYCP3.tif]

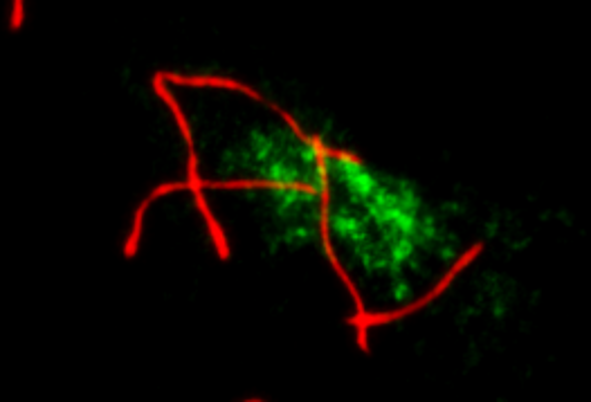

Supplement: Supplementary file 8 — Source data Fig. 4 [file 44318_2025_528_MOESM8_ESM.zip › Figure 4/4C/SyncSpdyacko-ChrY&SYCP3.tif]

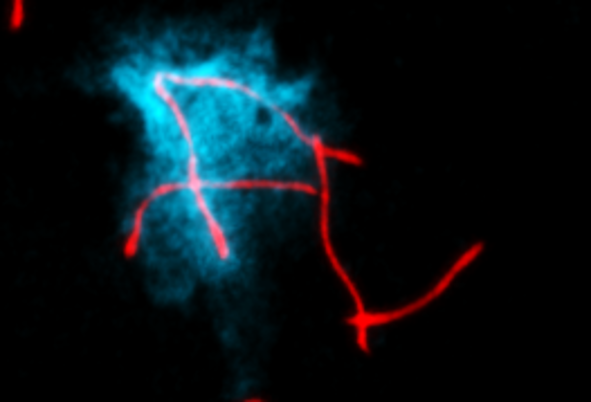

Supplement: Supplementary file 8 — Source data Fig. 4 [file 44318_2025_528_MOESM8_ESM.zip › Figure 4/4C/SyncSpdyacko-SCML2&SYCP3.tif]

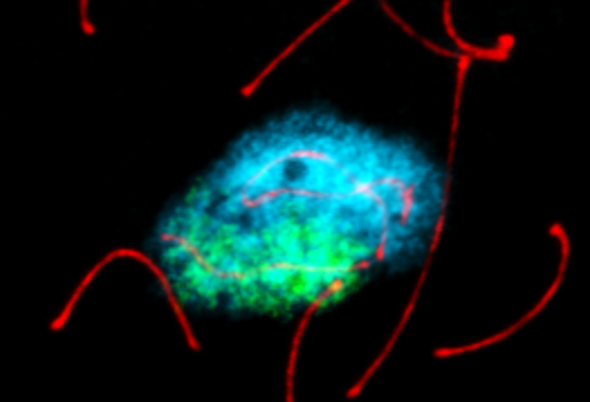

Supplement: Supplementary file 8 — Source data Fig. 4 [file 44318_2025_528_MOESM8_ESM.zip › Figure 4/4C/SyncSpdyaflfl-ChrY&SCML2&SYCP3.tif]

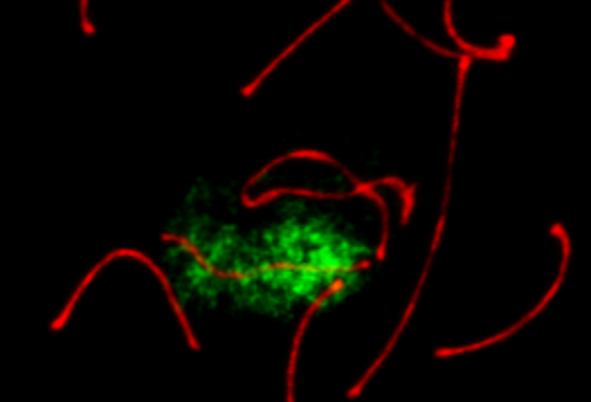

Supplement: Supplementary file 8 — Source data Fig. 4 [file 44318_2025_528_MOESM8_ESM.zip › Figure 4/4C/SyncSpdyaflfl-ChrY&SYCP3.tif]

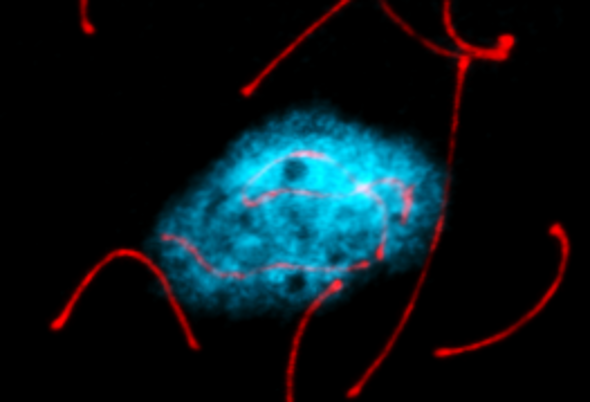

Supplement: Supplementary file 8 — Source data Fig. 4 [file 44318_2025_528_MOESM8_ESM.zip › Figure 4/4C/SyncSpdyaflfl-SCML2&SYCP3.tif]

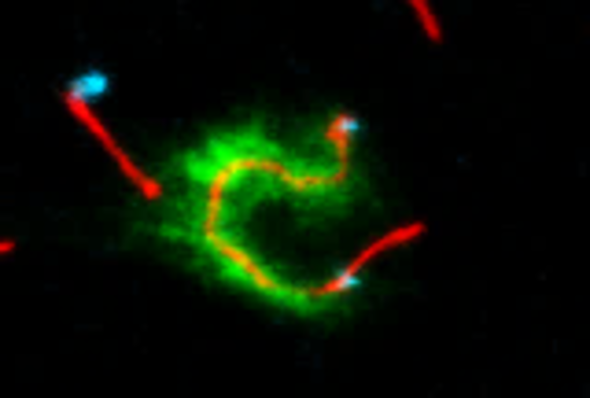

Supplement: Supplementary file 8 — Source data Fig. 4 [file 44318_2025_528_MOESM8_ESM.zip › Figure 4/4D/SyncSpdyacko-FK2&SYCP3&ACA.tif]

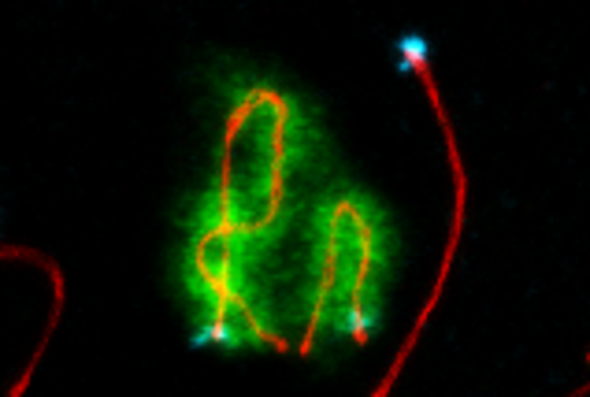

Supplement: Supplementary file 8 — Source data Fig. 4 [file 44318_2025_528_MOESM8_ESM.zip › Figure 4/4D/SyncSpdyaflfl-FK2&SYCP3&ACA.tif]

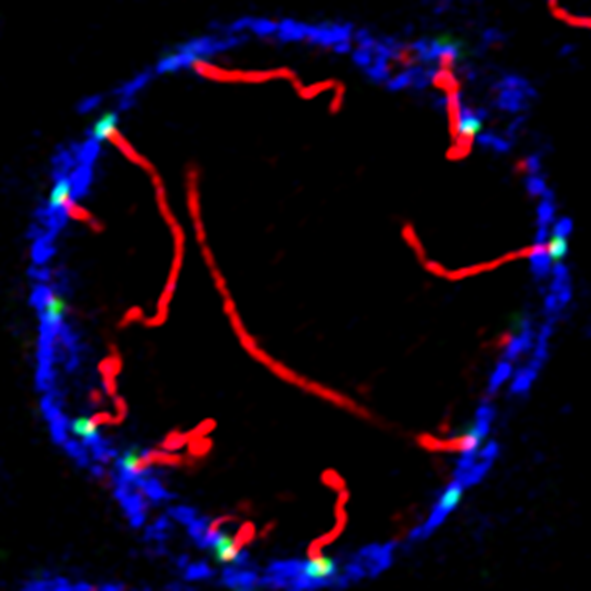

Supplement: Supplementary file 9 — Source data Fig. 5 [file 44318_2025_528_MOESM9_ESM.zip › Figure 5/5B/SyncSpdyacko-LaminB1&TRF1&SYCP1.tif]

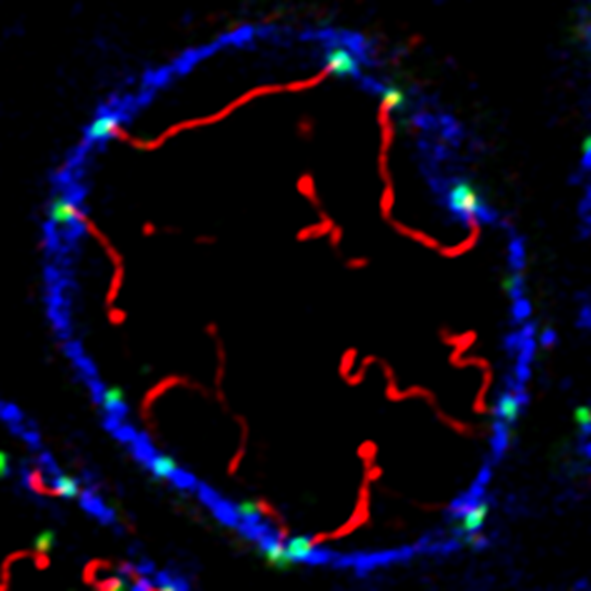

Supplement: Supplementary file 9 — Source data Fig. 5 [file 44318_2025_528_MOESM9_ESM.zip › Figure 5/5B/SyncSpdyaflfl-LaminB1&TRF1&SYCP1.tif]

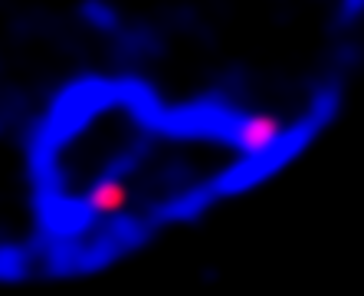

Supplement: Supplementary file 9 — Source data Fig. 5 [file 44318_2025_528_MOESM9_ESM.zip › Figure 5/5C/SyncSpdyacko-HORMAD1&LaminB1&ACA.tif]

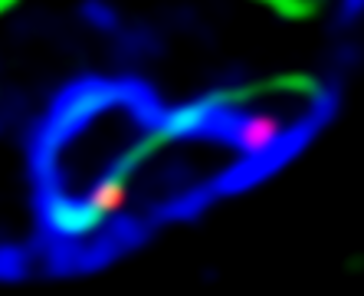

Supplement: Supplementary file 9 — Source data Fig. 5 [file 44318_2025_528_MOESM9_ESM.zip › Figure 5/5C/SyncSpdyacko-HORMAD1&LaminB1&SYCP1&ACA.tif]

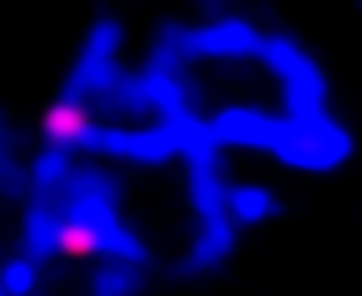

Supplement: Supplementary file 9 — Source data Fig. 5 [file 44318_2025_528_MOESM9_ESM.zip › Figure 5/5C/SyncSpdyaflfl-HORMAD1&LaminB1&ACA.tif]

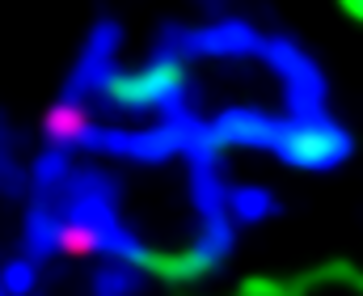

Supplement: Supplementary file 9 — Source data Fig. 5 [file 44318_2025_528_MOESM9_ESM.zip › Figure 5/5C/SyncSpdyaflfl-HORMAD1&LaminB1&SYCP1&ACA.tif]

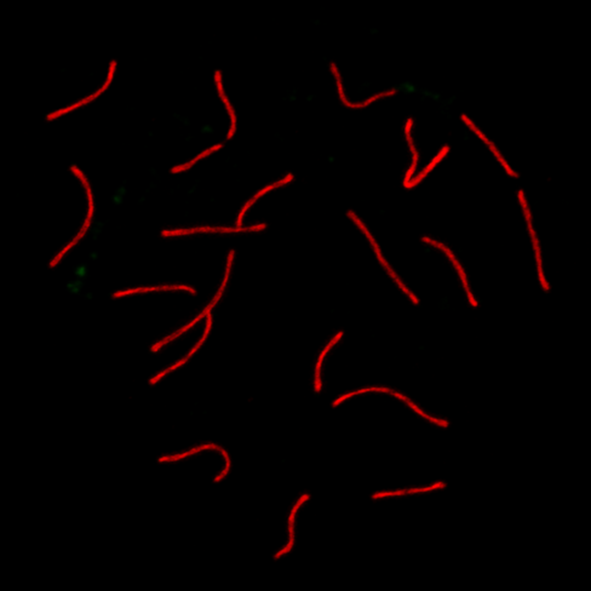

Supplement: Supplementary file 9 — Source data Fig. 5 [file 44318_2025_528_MOESM9_ESM.zip › Figure 5/5D/SyncSpdyacko-SUN1&SYCP3.tif]

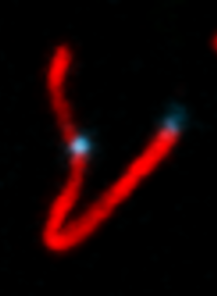

Supplement: Supplementary file 9 — Source data Fig. 5 [file 44318_2025_528_MOESM9_ESM.zip › Figure 5/5D/SyncSpdyacko-XY-SUN1&SYCP3&ACA.tif]

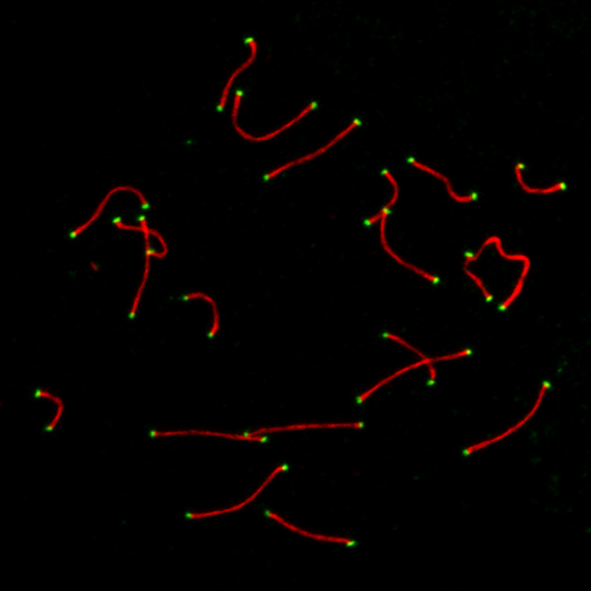

Supplement: Supplementary file 9 — Source data Fig. 5 [file 44318_2025_528_MOESM9_ESM.zip › Figure 5/5D/SyncSpdyaflfl-SUN1&SYCP3.tif]

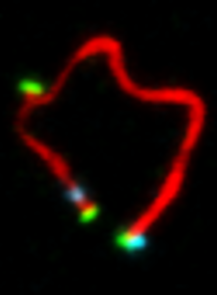

Supplement: Supplementary file 9 — Source data Fig. 5 [file 44318_2025_528_MOESM9_ESM.zip › Figure 5/5D/SyncSpdyaflfl-XY-SUN1&SYCP3&ACA.tif]

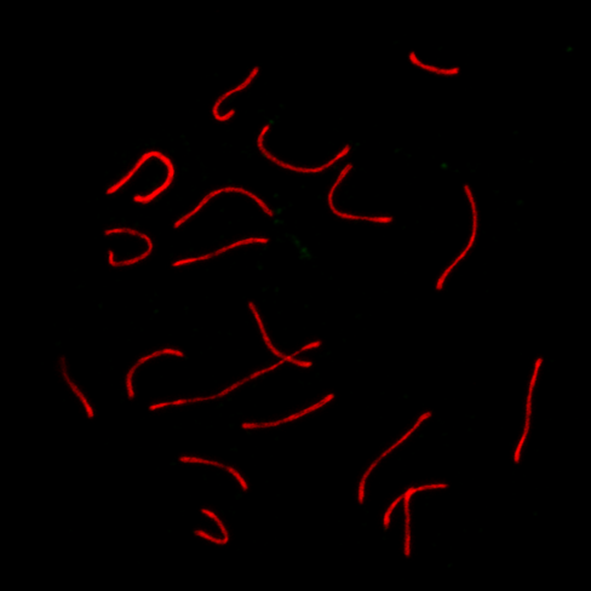

Supplement: Supplementary file 9 — Source data Fig. 5 [file 44318_2025_528_MOESM9_ESM.zip › Figure 5/5E/SyncSpdyacko-KASH5&SYCP3.tif]

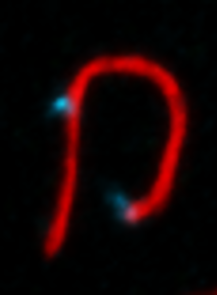

Supplement: Supplementary file 9 — Source data Fig. 5 [file 44318_2025_528_MOESM9_ESM.zip › Figure 5/5E/SyncSpdyacko-XY-KASH5&SYCP3&ACA.tif]

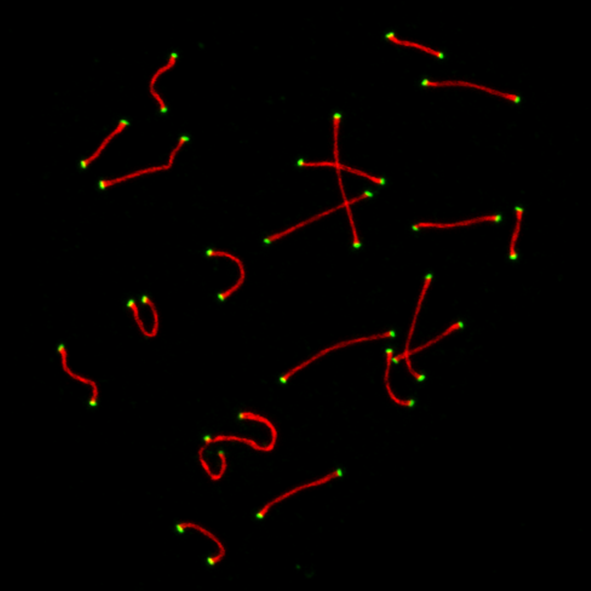

Supplement: Supplementary file 9 — Source data Fig. 5 [file 44318_2025_528_MOESM9_ESM.zip › Figure 5/5E/SyncSpdyaflfl-KASH5&SYCP3.tif]

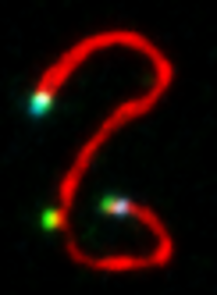

Supplement: Supplementary file 9 — Source data Fig. 5 [file 44318_2025_528_MOESM9_ESM.zip › Figure 5/5E/SyncSpdyaflfl-XY-KASH5&SYCP3&ACA.tif]

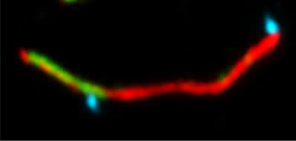

Supplement: Supplementary file 10 — Source data Fig. 6 [file 44318_2025_528_MOESM10_ESM.zip › Figure 6/6B/SyncTrf1cko-XY-SYCP1&SYCP3&ACA.tif]

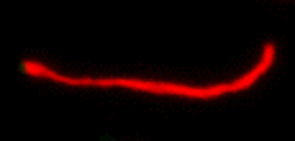

Supplement: Supplementary file 10 — Source data Fig. 6 [file 44318_2025_528_MOESM10_ESM.zip › Figure 6/6B/SyncTrf1cko-XY-SYCP3&TRF1.tif]

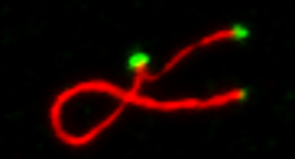

Supplement: Supplementary file 10 — Source data Fig. 6 [file 44318_2025_528_MOESM10_ESM.zip › Figure 6/6B/SyncTrf1flfl-XY-SYCP3&TRF1.tif]

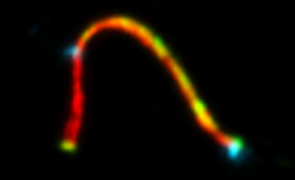

Supplement: Supplementary file 10 — Source data Fig. 6 [file 44318_2025_528_MOESM10_ESM.zip › Figure 6/6C/SyncTrf1cko-XY(a)-SpdyA&SYCP3&ACA.tif]

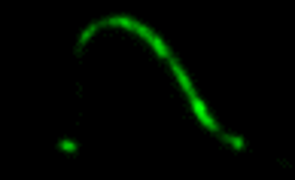

Supplement: Supplementary file 10 — Source data Fig. 6 [file 44318_2025_528_MOESM10_ESM.zip › Figure 6/6C/SyncTrf1cko-XY(a)-SpdyA.tif]

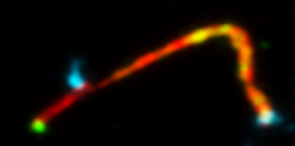

Supplement: Supplementary file 10 — Source data Fig. 6 [file 44318_2025_528_MOESM10_ESM.zip › Figure 6/6C/SyncTrf1cko-XY(b)-SpdyA&SYCP3&ACA.tif]

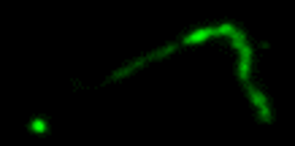

Supplement: Supplementary file 10 — Source data Fig. 6 [file 44318_2025_528_MOESM10_ESM.zip › Figure 6/6C/SyncTrf1cko-XY(b)-SpdyA.tif]

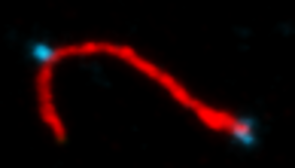

Supplement: Supplementary file 10 — Source data Fig. 6 [file 44318_2025_528_MOESM10_ESM.zip › Figure 6/6C/SyncTrf1cko-XY(c)-SpdyA&SYCP3&ACA.tif]

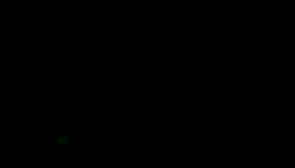

Supplement: Supplementary file 10 — Source data Fig. 6 [file 44318_2025_528_MOESM10_ESM.zip › Figure 6/6C/SyncTrf1cko-XY(c)-SpdyA.tif]

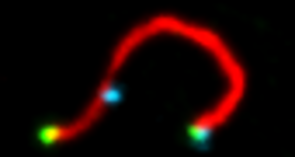

Supplement: Supplementary file 10 — Source data Fig. 6 [file 44318_2025_528_MOESM10_ESM.zip › Figure 6/6D/SyncTrf1cko-XY(a)-SUN1&SYCP3&ACA.tif]

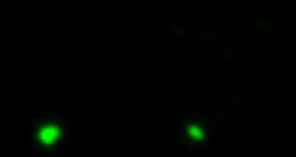

Supplement: Supplementary file 10 — Source data Fig. 6 [file 44318_2025_528_MOESM10_ESM.zip › Figure 6/6D/SyncTrf1cko-XY(a)-SUN1.tif]

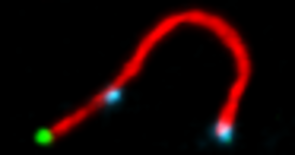

Supplement: Supplementary file 10 — Source data Fig. 6 [file 44318_2025_528_MOESM10_ESM.zip › Figure 6/6D/SyncTrf1cko-XY(b)-SUN1&SYCP3&ACA.tif]

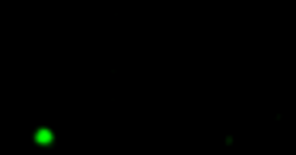

Supplement: Supplementary file 10 — Source data Fig. 6 [file 44318_2025_528_MOESM10_ESM.zip › Figure 6/6D/SyncTrf1cko-XY(b)-SUN1.tif]

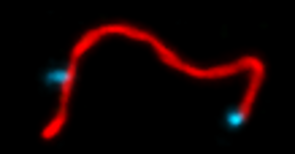

Supplement: Supplementary file 10 — Source data Fig. 6 [file 44318_2025_528_MOESM10_ESM.zip › Figure 6/6D/SyncTrf1cko-XY(c)-SUN1&SYCP3&ACA.tif]

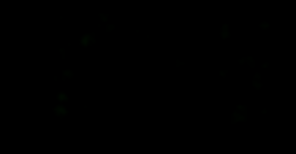

Supplement: Supplementary file 10 — Source data Fig. 6 [file 44318_2025_528_MOESM10_ESM.zip › Figure 6/6D/SyncTrf1cko-XY(c)-SUN1.tif]

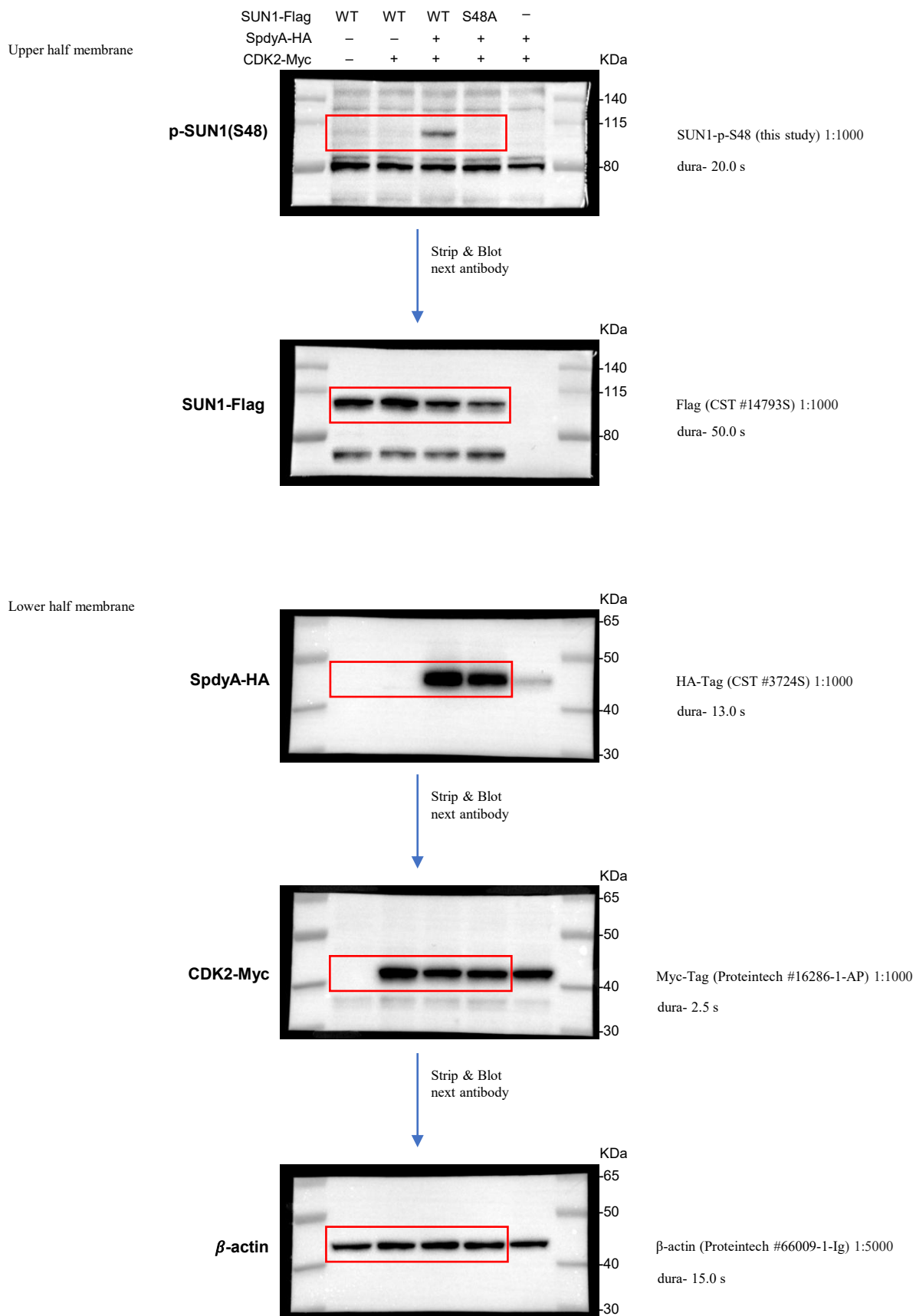

Supplement: Supplementary file 11 — Source data Fig. 7 [file 44318_2025_528_MOESM11_ESM.zip › Figure 7/7D/Immunoblotting membranes.pdf]

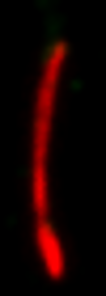

Supplement: Supplementary file 11 — Source data Fig. 7 [file 44318_2025_528_MOESM11_ESM.zip › Figure 7/7E/SyncSpdyacko-aut-pSUN1(S48)&SYCP3.tif]

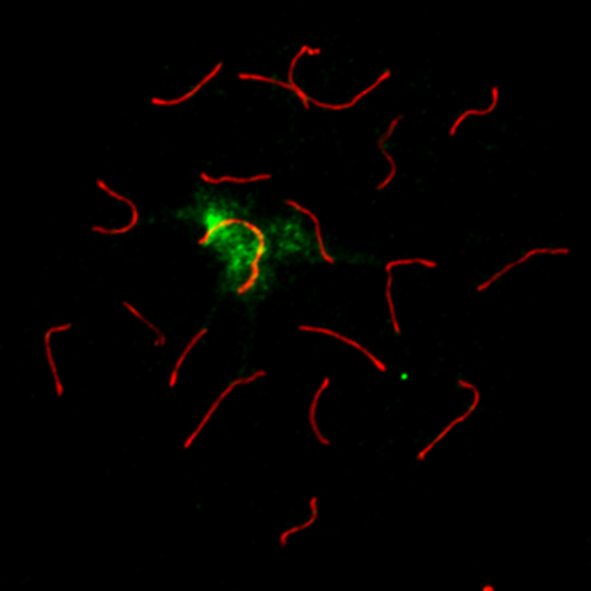

Supplement: Supplementary file 11 — Source data Fig. 7 [file 44318_2025_528_MOESM11_ESM.zip › Figure 7/7E/SyncSpdyacko-pSUN1(S48)&SYCP3.tif]

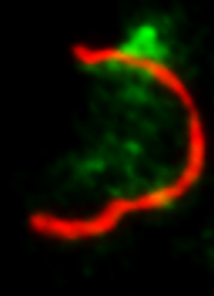

Supplement: Supplementary file 11 — Source data Fig. 7 [file 44318_2025_528_MOESM11_ESM.zip › Figure 7/7E/SyncSpdyacko-XY-pSUN1(S48)&SYCP3.tif]

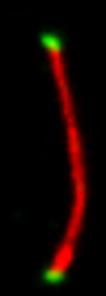

Supplement: Supplementary file 11 — Source data Fig. 7 [file 44318_2025_528_MOESM11_ESM.zip › Figure 7/7E/SyncSpdyaflfl-aut-pSUN1(S48)&SYCP3.tif]

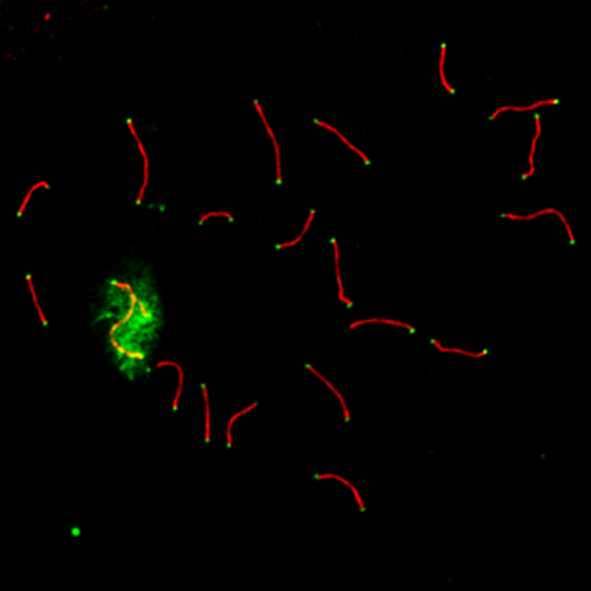

Supplement: Supplementary file 11 — Source data Fig. 7 [file 44318_2025_528_MOESM11_ESM.zip › Figure 7/7E/SyncSpdyaflfl-pSUN1(S48)&SYCP3.tif]

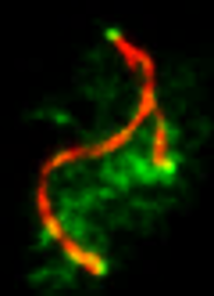

Supplement: Supplementary file 11 — Source data Fig. 7 [file 44318_2025_528_MOESM11_ESM.zip › Figure 7/7E/SyncSpdyaflfl-XY-pSUN1(S48)&SYCP3.tif]
