# Supplementary material for: Speedy A governs non-homologous XY chromosome desynapsis as a unique prerequisite for XY loop-axis organization
Source: EMBO J. 2025 Aug 18;44(19):5509–36. doi: 10.1038/s44318-025-00528-8 (PMC12488978; doi:10.1038/s44318-025-00528-8)

After the kinase reaction, the mixture was divided into two aliquots and loaded onto two separate SDS-PAGE gels.

Membrane 1

Membrane 2

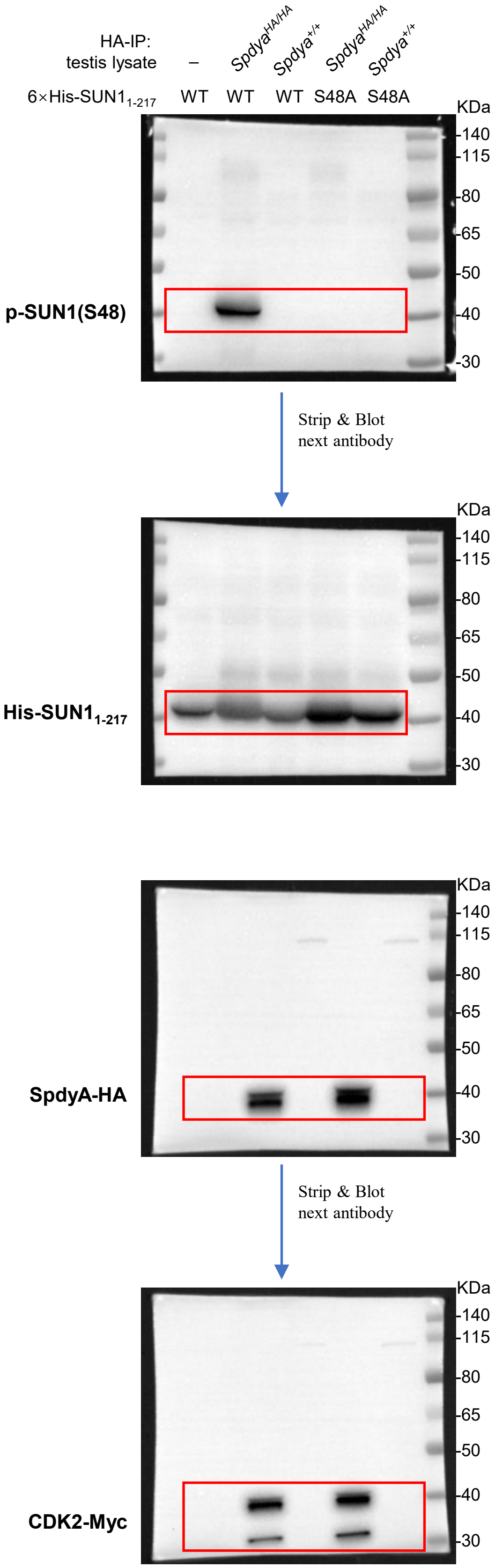

Supplement: Supplementary file 11 — Source data Fig. 7 [file 44318_2025_528_MOESM11_ESM.zip › Figure 7/7F/Immunoblotting membranes.pdf]
