# Supplementary material for: Speedy A governs non-homologous XY chromosome desynapsis as a unique prerequisite for XY loop-axis organization
Source: EMBO J. 2025 Aug 18;44(19):5509–36. doi: 10.1038/s44318-025-00528-8 (PMC12488978; doi:10.1038/s44318-025-00528-8)
Supplement: Supplementary file 12 — Expanded View Figures [file 44318_2025_528_MOESM12_ESM.pdf]

Expanded View Figures

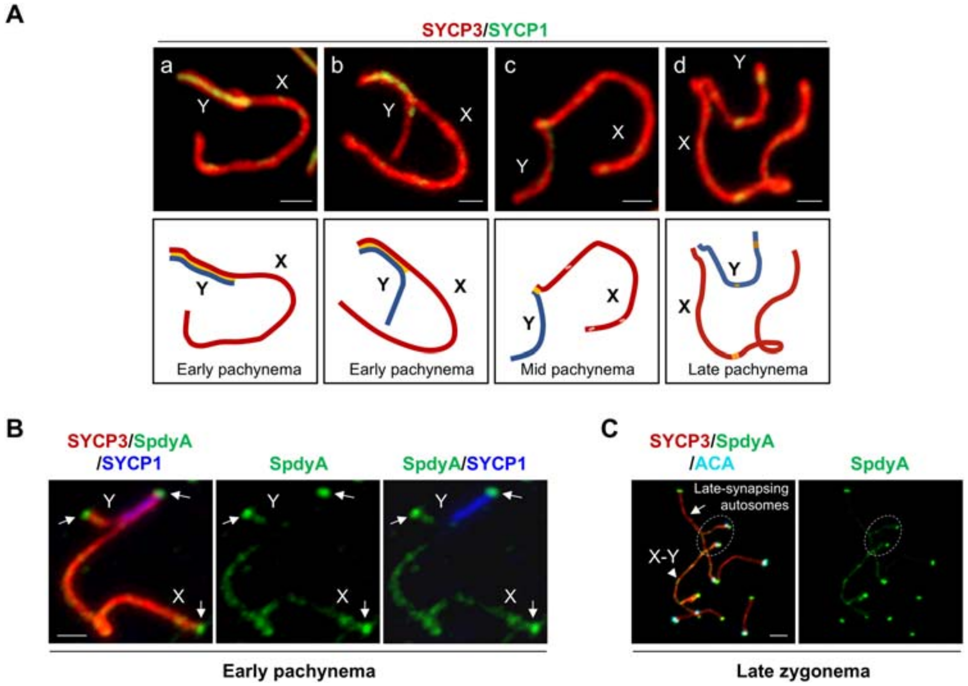

Figure EV1. Dynamic configuration changes of X and Y chromosomes and SpdyA localization at sex chromosome axes and telomeres during pachynema.

(**A**) Illustration of the dynamic changes in the X and Y chromosome configuration during pachynema. X and Y chromosomes were immunostained for SYCP3 (red) and SYCP1 (green). (a) At early pachynema, the X and Y chromosomes exhibit extensive side-by-side pairing, with the Y chromosomal axis fully aligned along the X chromosomal axis and with synaptonemal complex (SC) formation occurring between the aligned regions. This configuration is transient and rarely observed. (b) As pachynema progresses, the SC between the X and Y chromosomes gradually shortens. (c) By mid-pachynema, the SC becomes restricted to the PAR. (d) At late pachynema, desynapsis occurs and a chiasma forms, resulting in an "end-to-end" attachment between the X and Y chromosomes. Scale bars, 1 µm. (**B**) The localization of SpdyA at telomeres (arrows) and at the unsynapsed axes of X and Y chromosomes at early pachynema. X and Y chromosomes were immunostained for SYCP3 (red), SYCP1 (blue) and SpdyA (green). Scale bar, 1 µm. (**C**) SpdyA localization at the unsynapsed region (dashed circle) of late-synapsing autosomes at late zygonema. Scale bar, 2 µm.

 © The Author(s)

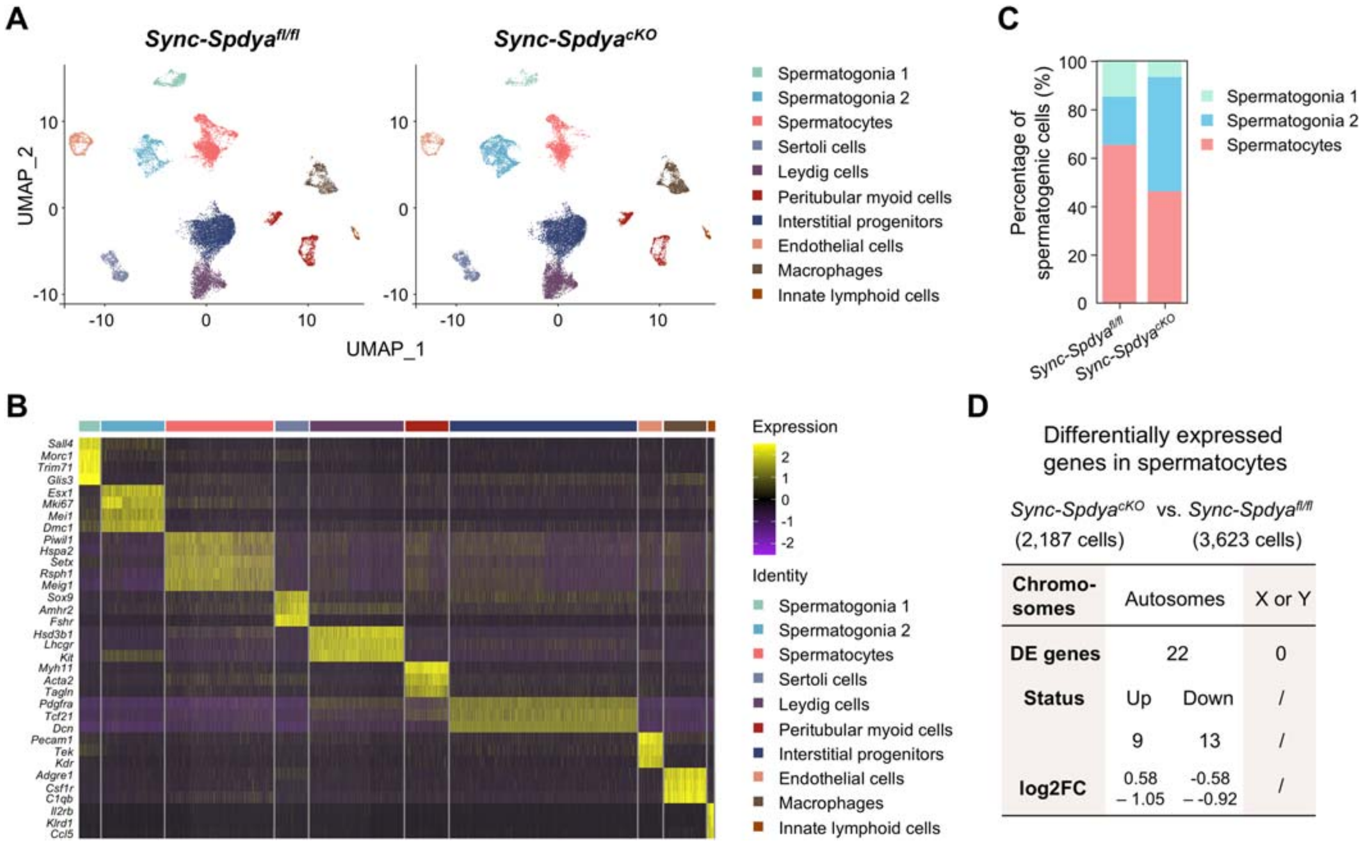

Figure EV2. Single-cell RNA-seq analysis revealed no differential expression of sex chromosome-linked genes in mid-late *Sync-Spdya^{cKO}* pachytene spermatocytes.

(**A**) UMAP and clustering analysis of single-cell transcriptome data from testicular cells collected at RA11.75 from tamoxifen-treated *Spdya$^{fl/fl}$* and *Spdya$^{fl/fl}$;Ddx4-Cre^{ERT2}* mice. Each dot represents a single cell, colored according to its cluster identity. UMAP, Uniform Manifold Approximation and Projection. (**B**) Heatmap showing expression of marker genes for 10 identified cell types (right). The pachytene cell marker *Hspa2* is highly expressed in spermatocytes. Spermatogonia 1 and 2 likely represent undifferentiated and differentiating spermatogonia, respectively, based on their marker gene profiles. (**C**) Proportion of the three types of spermatogenic cell types. (**D**) Differential expression analysis of 2187 *Sync-Spdya^{cKO}* and 3623 *Sync-Spdya$^{fl/fl}$* pachytene spermatocytes. DE genes, differentially expressed genes; Log2FC, $\log_2$ fold change. Cutoff criteria: $|\log2FC| \geq 0.58$ and adjusted P value < 0.05.

© The Author(s)

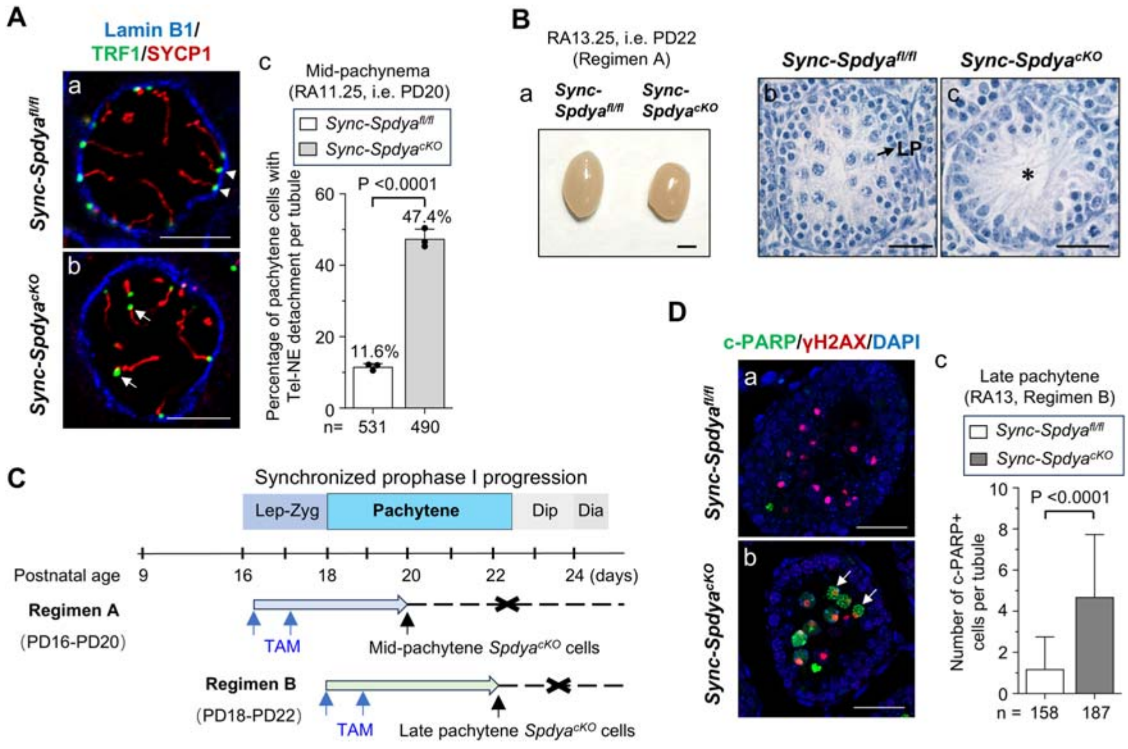

Figure EV3. SpdyA is essential for spermatocyte survival throughout pachynema.

(**A**) Compared to *Sync-Spdya*$^{fl/fl}$ cells (a), mid-pachytene (RA11.25) *Sync-Spdya*^{cKO} cell (b) exhibited severe Tel-NE detachment (arrows). Testis sections were immunostained for TRF1 (green), SYCP1 (red) and Lamin B1 (blue). Arrowheads indicate Tel-NE attachment. Scale bars, 5 µm. (c) Percentages of pachytene cells with Tel-NE detachment in mid-pachytene *Sync-Spdya*$^{fl/fl}$ and *Sync-Spdya*^{cKO} testes at RA11.25. "*n*" represents the total number of pachytene spermatocytes scored from three mice per genotype. (**B**) Following tamoxifen regimen shown in Fig. 2A, the *Sync-Spdya*^{cKO} testes collected at RA13.25 were smaller in size and lacked late pachytene spermatocytes. LP, late pachytene spermatocyte; asterisk indicates loss of spermatocytes. Scale bars, 30 µm. (**C**) Summary of tamoxifen treatment regimens used to obtain mid- and late pachytene *Sync-Spdya*^{cKO} spermatocytes. Regimens A and B correspond to those shown in Figs. 2A and 3A, respectively. Dashed lines with crosses indicate stages at which *Sync-Spdya*^{cKO} spermatocytes could not be obtained. (**D**) Following Regimen B, late pachytene *Sync-Spdya*^{cKO} spermatocytes collected at RA13 exhibited apoptosis. Testis sections were immunostained for c-PARP (green) and γH2AX (red). Nuclei were counterstained with DAPI. Scale bars, 30 µm. "*n*" represents the total number of seminiferous tubules scored from three mice per genotype. Data information: All values are presented as the mean ± SD. Statistical analyses were performed using two-tailed Student's *t* test in (**A**) with $P = 0.000025$, and Mann–Whitney test in (**D**) with $P < 0.000000000000001$.

 © The Author(s)

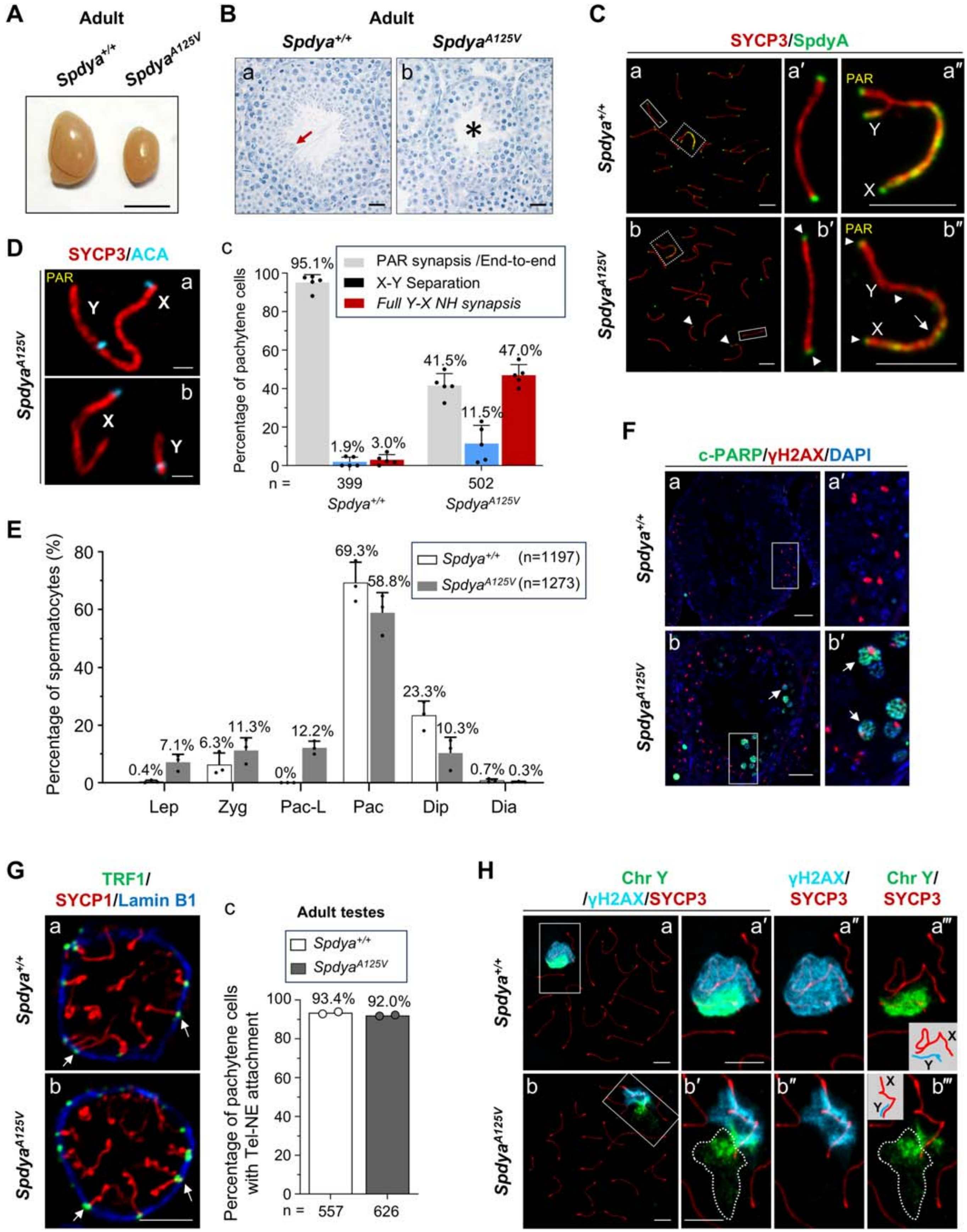
A
Adult
Spdya+/+
SpdyaA125V
B
Adult
Spdya+/+
SpdyaA125V
C
SYCP3/SpdyA
PAR
Y
X
D
SYCP3/ACA
c
PAR synapsis /End-to-end
X-Y Separation
Full Y-X NH synapsis
Percentage of pachytene cells
95.1%
1.9%
3.0%
41.5%
11.5%
47.0%
n = 399 502
E
Percentage of spermatocytes (%)
Spdya+/+ (n=1197)
SpdyaA125V (n=1273)
0.4%
7.1%
6.3%
11.3%
0%
12.2%
69.3%
58.8%
23.3%
10.3%
0.7%
0.3%
Lep
Zyg
Pac-L
Pac
Dip
Dia
F
c-PARP/γH2AX/DAPI
G
TRF1/
SYCP1/Lamin B1
c
Adult testes
Percentage of pachytene cells with Tel-NE attachment
93.4%
92.0%
n = 557 626
H
Chr Y
/γH2AX/SYCP3
γH2AX/
SYCP3
Chr Y/
SYCP3

Figure EV4. Adult $Spdya^{A125V}$ mice is a hypomorphic-SpdyA model in which pachytene spermatocytes exhibited *full Y-X NH synapsis* and apoptosis.

(**A**) The size of testis was smaller in the 3-month-old (adult) $Spdya^{A125V}$ mice. Scale bar, 5 mm. (**B**) Histological analyses of testes from adult $Spdya^{+/+}$ (a) and $Spdya^{A125V}$ (b) mice. Red arrow indicates great amounts of elongated spermatids in $Spdya^{+/+}$ seminiferous tubule, but they were difficult to find in $Spdya^{A125V}$ seminiferous tubule (asterisk). Scale bars, 30 µm. (**C**) SpdyA signal intensity was strikingly reduced at telomeres (b–b″, arrowheads) and sex chromosome axes (b″, arrow) in $Spdya^{A125V}$ pachytene spermatocyte. Magnified views (a′–a″, b′–b″) are indicated by solid and dashed rectangles in (a–b). Scale bars, 5 µm. (**D**) Numbers of $Spdya^{A125V}$ pachytene cells exhibited *full Y-X NH synapsis* (a) and X-Y separation (b). Scale bars, 1 µm. (c) Percentages of pachytene cells with different X-Y configurations in testes from $Spdya^{+/+}$ and $Spdya^{A125V}$ adult male mice. (**E**) Analyses of meiotic stages in spermatocytes from adult $Spdya^{+/+}$ and $Spdya^{A125V}$ mice, based on immunostaining of chromosome spreads. Lep, leptotene; Zyg, zygotene; Pac-L, pachytene-like; Pac, pachytene; Dip, diplotene; Dia, diakinesis spermatocytes. (**F**) IF analyses of $Spdya^{+/+}$ (a–a′) and $Spdya^{A125V}$ (b–b′) testis sections immunostained for c-PARP (green) and γH2AX (red). Solid rectangles indicate areas magnified in (a′–b′). Arrows indicate apoptotic spermatocytes. Scale bars, 30 µm. (**G**) IF analyses of $Spdya^{+/+}$ and $Spdya^{A125V}$ testis sections with Lamin B1 (blue), TRF1 (green) and SYCP1 (red). Arrows indicate Tel-NE attachment. Scale bars, 5 µm. (c) Percentages of $Spdya^{+/+}$ and $Spdya^{A125V}$ pachytene cells with intact Tel-NE attachment. (**H**) Sex chromosomes in late pachytene $Spdya^{A125V}$ spermatocytes with *full Y-X NH synapsis* displayed disrupted loop-axis organization. Chromosome spreads were subjected to immuno-FISH staining for γH2AX (light blue), Chr Y (green) and SYCP3 (red). Magnified views (a′–a‴, b′–b‴) are indicated by solid rectangles in (a, b). White dashed line-enclosed area indicates Y chromatin signals located outside the γH2AX area. Scale bars, 5 µm. Data information: In (**D**, **E**), values are presented as the mean ± SD; In (**G**), data are presented as the mean with individual values. "*n*" represents the total number of pachytene spermatocytes scored per genotype: from five mice in (**D**), from three mice in (**E**), and from two mice in (**G**).

 © The Author(s)
